# Mechanism of the Low-Temperature Organic Removal from Imidazolium-Containing Zeolites by Ozone Treatment: Fluoride Retention in Double-4-Rings

**DOI:** 10.1021/acs.inorgchem.4c01021

**Published:** 2024-05-17

**Authors:** Zihao Rei Gao, Carlos Márquez-Álvarez, Salvador R. G. Balestra, Huajian Yu, Luis A. Villaescusa, Miguel A. Camblor

**Affiliations:** † Instituto de Ciencia de Materiales de Madrid (ICMM), CSIC, c/Sor Juana Inés de la Cruz 3, 28049 Madrid, Spain; ‡ Instituto de Catálisis y Petroleoquímica (ICP), CSIC, c/Marie Curie 2, 28049 Madrid, Spain; § Centro de Nanociencia y Tecnologías Sostenibles (CNATS), Departamento de Sistemas Físicos, Químicos y Naturales, Universidad Pablo de Olavide, Ctra. Utrera Km 1, ES-41013 Seville, Spain; ∥ Instituto Interuniversitario de Investigación de Reconocimiento Molecular y Desarrollo Tecnológico (IDM) Universitat de València−Universitat Politècnica de València, Camino de Vera s/n, 46022 Valencia, Spain; ⊥ CIBER de Bioingeniería Biomateriales y Nanomedicina (CIBER-BBN), 28029 Madrid, Spain; # Departamento de Química, Universitat Politècnica de València, Camí de Vera s/n, 46022 Valencia, Spain

## Abstract

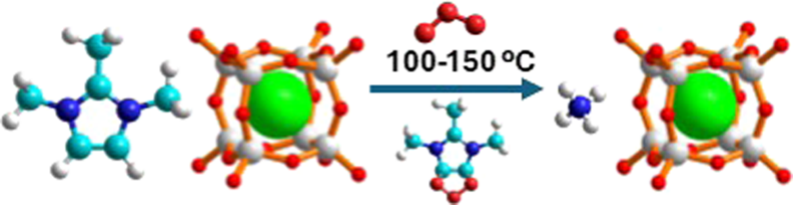

For zeolites synthesized using imidazolium cations, the organic matter can be extracted at very low temperatures (100 °C) using ozone. This is possible for zeolites with 12-ring or larger pores but requires higher temperatures in medium-pore zeolites. The first chemical events in this process occur fast, even at room temperature, and imply the loss of aromaticity likely by the formation of an adduct between ozone and the imidazole ring through carbons C4 and C5. Subsequent rupture of the imidazole ring provides smaller and more flexible fragments that can desorb more readily. This process has been studied experimentally, mainly through infrared spectroscopy, and theoretically by density functional theory. Amazingly, fluoride anions occluded in the small double-four-ring units (*d4r*) during the synthesis remain inside the cage throughout the whole process when the temperature is not too high (≤150 °C). However, fluoride in larger cages in **MFI** ends up bonded to silicon in penta or hexacoordinated units, likely out of the cages, after ozone treatment at 150 °C. For several germanosilicate zeolites, the process allows their subsequent degermanation to yield stable high-silica zeolites. Quaternary ammonium cations require harsher conditions that eventually also extract fluoride from zeolite cages, including the *d4r* unit.

## Abstract 2

Fluoride is retained in double-4-ring cages after detemplation of imidazole-templated zeolites with ozone at mild temperatures (100−150 °C). We unveil the mechanism of ozonolysis by an experimental–theoretical approach.

## 1 Introduction

Zeolites are microporous crystalline materials that are frequently synthesized with the aid of organic additives that act as “organic structure directing agents,” OSDAs, and are finally retained in the as-synthesized materials, rendering them nonporous unless these organics are removed. The removal of those organic molecules to open the micropores is generally attained by calcination in air at relatively high temperatures, typically above 400 or 500 °C. This is obviously energetically costly and economically expensive and may cause structural damage and the extraction of certain heteroatoms (atoms other than Si) from the framework. In the case of zeolite membranes, calcination may introduce cracks that compromise their separation performance and mechanical properties.^1^ Thus, there is a significant interest in developing routes for the removal of organics from zeolites through milder treatments.^2^ The effectiveness of such methods depends on the pore system and on the nature of the OSDA. Neutral triethylamine could be removed from AlPO-5 by treatment with methanolic hydrochloric acid at 147 °C.^3^ Organic quaternary tetraethylammonium was removed from the large-pore zeolite beta by a hydrothermal treatment with HNO_3_ but at the cost of extracting also Al from the framework, which diminishes the crystallinity and reduces the number of catalytic sites.^4^ Alkylammonium cations could also be removed from zeolites at low temperature by a dry air,^5^ or oxygen radiofrequency plasma,^6^ possibly with the intervention of O_3_ and excited oxygen species, as it occurs in the detemplation by dielectric barrier discharge plasma.^7^

Although the elimination of carbonaceous deposits (i.e., coke) from spent zeolite catalysts using O_3_ dates back to the mid 1980s,^8^ the low-temperature OSDA removal by O_3_ treatments was first specifically reported in 1994 when van der Waals et al. synthesized pure silica zeolite β using dibenzyldimethylammonium and detemplated it by a treatment at 110 °C in an O_3_ stream followed by washing with hot acetone.^9^ The motivation for that treatment was to avoid the formation of difficult-to-remove coke when the zeolite was calcined in air at high temperature.^10^ Despite the considerable diffusion of that paper (it was the first report on the long sought synthesis of pure silica β), the O_3_ treatment passed quite unnoticed for several years, and, in fact, none of the first several subsequent papers on the O_3_-mediated removal of the OSDA (frequently called detemplation) has cited this seminal work, to our surprise. Next ozone detemplation reports concerned mesoporous materials rather than zeolites.^11^ With the new century, the use of O_3_ to remove the occluded OSDA was “rediscovered.” For instance, Gilbert et al. reported the removal of tetrapropylammonium (TPA) from silicalite-1 (**MFI**), although at temperatures rather high (500 °C for the total removal).^12^ However, Heng et al. used O_3_ at 200 °C to remove TPA from **MFI** membranes, concluding that the time needed for total removal depends on the membrane thickness, O_3_ concentration, and Al content and showing important benefits for the membrane performance compared to standard calcination.^13^ In all of these and in subsequent reports, the OSDA removed were quaternary ammonium cations or neutral alkylamines.^14^

In recent years, imidazolium cations have received much attention as nonquaternary ammonium OSDA cations in the synthesis of zeolites.^15^ Although initial studies only produced known zeolites,^16^ imidazolium OSDAs have later afforded the synthesis of silica (ITQ-12,^17^ HPM-1^18,19^), aluminosilicate (**RTH**,^20−22^**PWO**, **PWW**,^23,24^ PST-24^25^), and germanosilicate zeolites (NUD-1,^26^ NUD-2,^27^ NUD-3,^28^ CIT-13,^29^ HPM-7,^30^ HPM-8,^31^ HPM-12,^32^ HPM-14,^33^ HPM-16,^34^ and PST-35^35^), sometimes with totally new structures. The structural richness of germanosilicate zeolites is, however, counterbalanced by the fact that four-coordinated germanium atoms in a zeolite framework are typically prone to hydrolysis and framework extraction by contact with moisture after calcination. Although in some cases, it is possible to take profit out of the instability of germanium atoms in zeolites to develop new materials, as in the ADOR process,^36^ in many cases, it instead limits the practical applications of these new zeolite structures.

However, as we recently reported, O_3_ detemplation of HPM-16 (-**HOS**), a large/medium-pore germanosilicate zeolite with an interrupted framework, allowed its subsequent degermanation to yield a stable quasi-silica zeolite. Here, we report our experimental and theoretical investigation of the low-temperature O_3_-mediated organics removal from imidazolium-based zeolites, which provides some insights into the molecular mechanism and demonstrates the remarkable fact that fluoride anions occluded in double-four-rings (*d4r*) during the synthesis remain in the zeolite when the detemplation temperature is not high. When this work was about to be submitted, a paper by the groups of Auerbach and Fan on a similar topic but with a different focus was published online.^37^ There, the authors reported the removal of imidazolium from an LTA zeolite by an ozone treatment at 175 °C. Fluoride was also removed from the *d4r* cages but remained somewhere else in the zeolite. In our work,^38^ which does not contradict the general conclusions drawn in the paper mentioned, we focus instead on the mechanism of imidazolium degradation, on fluoride retention as a possible way to develop a new kind of acid zeolite, and on the possibility to degermanate silicogermanate zeolites detemplated by ozone treatment in order to obtain stable zeolites. Traditional acidic zeolites, which have found and enormous variety of applications in catalysis, rely on aluminosilicate zeolites with protons as counterbalance cations. The acid site in that case is a proton close to a [AlO_4_]^−^ unit. The possibility to develop a material in which the acid center is a proton further apart from the negative charge and shielded from it (such as H^+^F@*d4r*, i.e., a proton counterbalancing a fluoride that is inside a SiO_2_*d4r*) is, in our opinion, highly attractive, at least from a fundamental point of view.

## 2 Experimental Section

### 2.1 Synthesis of Zeolites

All of the zeolites used in this article, listed in Table 1, have been prepared according to our previously published procedures, except silica beta prepared with 1,1′-(butane-1,4-diyl)bis(3-benzyl-2-methylimidazolium) (4bBnMI), whose synthesis is described below. Nine zeolites were prepared with imidazolium compounds, while one was prepared using a quaternary ammonium OSDA for comparison.

**Table 1:** Structural Properties, T Atoms, Crystal Size, and OSDAs of the Zeolites Studied in This Work

zeolite	ZFT^a^	channels^b^	T atoms	crystal size (μm)	OSDA	refs
HPM-1	**STW**	10 × 8 × 8 MR	Si	2–20	2-ethyl-1,3,4-trimethylimidazolium (2E134TMI)	(18)
HPM-7	**POS**	12 × 11 × 11 MR	Ge,Si	4 × 0.1 × 0.1	1,1′-(decane-1,10-diyl)bis(2,3-dimethylimidazolium) (10BDMI)	(31)
HPM-8		12 × 12 × 12 MR	Ge,Si	0.5 × 0.5 × 0.5	1,1′-(decane-1,10-diyl)bis(2,3-dimethylimidazolium) (10BDMI)	(31)
HPM-14		16 × 9 × 9 MR	Ge,Si	10 × 0.2 × 0.2	1-methyl-3-(2′,4′,6′-trimethylbenzyl)imidazolium (1M3TMBzI)	(33)
HPM-16	**-HOS**	12 × 10 × 12 MR	Ge,Si	4 × 1 × 0.15	1-methyl-2-ethyl-3-*n*-propylimidazolium (1M2E3nPrI)	(34)
SYSU-3	**-SYT**	24 × 8 × 8 MR	Ge,Si	1 × 0.15 × 0.15	1-methyl-2-ethyl-3-*n*-propylimidazolium (1M2E3nPrI)	(39)
	**MFI**	10 × 10 MR	Si	1 × 1 × 0.3	1,1′-(butane-1,4-diyl)bis(3-methylimidazolium) (4BI)	(40)
ITQ-7	**ISV**	12 × 12 × 12 MR	Si	0.5 × 0.5 × 0.2	1,3,3-trimethyl-6-azoniumtricyclo3.2.1.4^6,6^dodecane (EABO)	(41)
beta		12 × 12 × 12 MR	Si	0.75 × 0.3 × 0.3	1,1′-(octane-1,8-diyl)bis(2,3-dimethylimidazolium) (8bDMI)	(31)
beta		12 × 12 × 12 MR	Si	0.7 × 0.1 × 0.1	1,1′-(butane-1,4-diyl)bis(3-benzyl-2-methylimidazolium) (4bBnMI)	this work

a ZFT are zeolite framework type codes assigned by the Structure Commission of the International Zeolite Association (SC-IZA) to confirmed ordered zeolite structures.^42^ HPM-8 and HPM-14 have no assigned codes. HPM-8 is an intergrowth of polymorphs D and E of the zeolite beta family, with over 80% of monoclinic polymorph D. HPM-14 is an intergrowth of two polymorphs with 85–90% predominance of the monoclinic polymorph. The *BEA code for beta has been discontinued by the IZA.

b Number of tetrahedra of the window limiting diffusion along the largest pore in different directions.

#### 2.1.1 Synthesis of 4bBnMI-SiO_2_–Beta Zeolite

##### 2.1.1.1 Synthesis of the OSDA

1-Benzyl-2-methylimidazole (120 mmol, 20.67 g, Sigma-Aldrich 90%) and 1,4-Dibromobutane (50 mmol, 10.80 g, Sigma-Aldrich 99%) were added dropwise to 150 mL of acetonitrile (Scharlab). After 4 days of refluxing and stirring, the solid product was obtained by filtration and then washed with dimethyl ether. After drying at room temperature, 1,1′-(butane-1,4-diyl)bis(3-benzyl-2-methylimidazolium) (4bBnMI) bromide was obtained (yield: 70%). The purity of the product was confirmed by ^1^H and ^13^C NMR spectra by dissolving the bromide salts in D_2_O. ^1^H NMR (300 MHz, D_2_O) δ 7.55–7.37 (m, 10H), 7.33 (dt, *J* = 6.8, 2.2 Hz, 4H), 5.37 (s, 4H), 4.27–4.13 (m, 4H), 2.57 (s, 6H), 1.84 (p, *J* = 3.3 Hz, 4H). ^13^C NMR (75 MHz, D2O) δ 144.15, 133.66, 129.33, 128.97, 127.79, 121.78, 121.15, 51.51, 47.43, 25.83, 9.33.

To obtain the OSDA in hydroxide form, 17.73 g 4bBnMI bromide was added to a mixture of 240 mL of pure water and 120 mL of anion exchange resin (Amberlite IRN78 OH hydroxide from, Sigma-Aldrich; exchange capacity: 1.1 mmol per mL wet resin). After stirring overnight, the OSDAOH solution was collected by filtration. The diluted OSDA hydroxide solution was concentrated in a rotary evaporator at 72 °C, and the concentration of solution was determined by titration with 0.1 M HCl solution (exchange ratio 91%).

##### 2.1.1.2 Synthesis of the Zeolite

The zeolite was synthesized from a gel of composition 0.5 OSDAOH/0.5 HF/1 SiO_2_/5 H_2_O at 150 °C in a rotary oven for 16 days. 4.1666 g (20.00 mmol) of tetraethyl orthosilicate (Sigma-Aldrich, 99%) was added to 34.0599 g OSDAOH (10.00 mmol, [OH^–^] = 0.2936 mmol/g). After stirring overnight for hydrolysis, when the target water amount was reached, 362 μL of HF (9.99 mmol, 27.6 mol/L) was added, and then the gel was separated into four parts and transferred to four 30 mL Teflon insert autoclaves. After crystallization, the product was filtered and washed with water (25 mL × 2) and acetone (25 mL × 1), and the solid was dried at RT. **Caution!** HF is irritant, corrosive, and toxic. Work under a hood and wear the appropriate personal protection equipment.

### 2.2 O_3_ Treatments

Detemplation of the zeolite samples was carried out by a low-temperature thermal treatment under an ozone–oxygen mixture flow. Ozone was produced from high-purity oxygen (≥99.995 vol %) at a rate of 500 mg/h using a Salveco Proyectos ECO_3_ C-3 electrical discharge ozone generator. **Caution!** Ozone can cause severe respiratory damage and skin and eye irritation. Also, unknown organics can evolve during the experiment. Work under a hood, and use an ozone decomposer before releasing the gases to the atmosphere.

### 2.3 Degermanation

Degermanation of the germanosilicate zeolites in Table 1 was performed in acidic conditions inspired by methods previously reported in the literature.^43,44^ HPM-7 possesses the **POS** structure,^30^ HPM-16 (**-HOS**)^34^ was recently reported as containing 12 × 10(12) × 12 pores, and HPM-8 is a 12-ring zeolite of the beta family consisting of an intergrowth of polymorphs D and E, with a very large predominance of polymorph D.^31^ For HPM-16, the precise degermanation method has been already reported and applied on an O_3_ treated sample.^34^ For HPM-7, a similar procedure was followed while the degermanation on HPM-14 and SYSU-3 failed to obtain stable zeolite phases. In the case of HPM-8, degermanation was carried out on both an O_3_ treated sample and a sample calcined in standard high-temperature conditions for comparison. First, 210.2 mg of the samples were suspended into 15 mL of HCl aqueous solution (1 M), with the addition of 75 μL of TEOS as the silicon source; second, the mixture was stirred for 30 min and then transferred into an autoclave within a 30 mL Teflon insert, followed by a hydrothermal treatment at 120 °C for 1 day; finally, after the hydrothermal treatment, the solid product was collected by filtration and washed with water (30 mL × 3) and acetone (30 mL × 1). Between one and three identical degermanation steps were performed in order to obtain highly stable quasi-pure-silica degermanated HPM-8 zeolites.

### 2.4 Characterization

Powder X-ray diffraction (PXRD) patterns were collected on a Bruker D8 Advanced diffractometer equipped with a Cu Kα radiation source. Elemental analysis of C, H, and N was carried out on a LECO CHNS-932 machine. Thermogravimetric analysis (TGA) experiments were performed on a SDT Q600 TA Instrument under airflow (100 mL/min) from 25 to 1000 °C at 10 °C/min. Field emission scanning electron microscopy (FE-SEM) and energy-dispersive X-ray spectroscopy (EDS) experiments were carried on an FEI Nova NanoSEM 230 machine equipped with a Genesis XM2i EDS detector. Magic angle spinning (MAS) nuclear magnetic resonance (NMR) spectra (^1^H, ^13^C, ^19^F, ^29^Si) were collected on a Bruker AV-400-WV equipment, and detailed experimental parameters have been given elsewhere.^45^

Attenuated total reflection Fourier transform infrared spectroscopy (ATR-FTIR) was used to follow the kinetics of the ozone treatments. ATR-FTIR spectra of as-synthesized samples and samples treated under ozone for selected time periods were recorded using a Nicolet Nexus 670 spectrometer provided with a GladiATR single-bounce monolithic diamond ATR accessory and an MCT cryodetector. The spectra were recorded in the 4000–650 cm^–1^ range, at 4 cm^–1^ resolution, by averaging 128 scans. FTIR spectra of the samples in transmission mode using the KBr pellet method were recorded in a Bruker Vertex 70 V spectrophotometer at a resolution of 2 cm^–1^ with 100 scans per sample. FTIR spectra of self-supporting wafers were recorded using an all-glass transmission cell provided with ZnSe windows and connected to a vacuum line. Samples were pressed into self-supporting wafers of ca. 4 mg cm^–2^ thickness and degassed at selected temperatures (pressure below 10^–4^ hPa). Temperature-programmed desorption (TPD-FTIR) analysis was performed by collecting spectra periodically while increasing the sample wafer temperature at a rate of 2 °C/min under dynamic vacuum. Spectra were recorded in the 4000–650 cm^–1^ wavenumber range, at 4 cm^–1^ resolution, by averaging 128 scans (total collection time ca. 0.5 min per spectrum) using a Thermo Nicolet Nexus 670 FTIR spectrophotometer equipped with an MCT cryodetector.

A temperature-programmed desorption study of ozone-treated samples with analysis of the evolved gaseous products by mass spectrometry (TPD-MS) was carried out using a Pfeiffer Vacuum QME 220 quadrupolar mass spectrometer. Samples were introduced in a quartz tube connected to the turbomolecular pump of the spectrometer, the tube was evacuated at room temperature and, subsequently, the temperature was increased at a rate of 10 °C/min. Mass spectra of the evolved gases were acquired in multiple ion detection (MID) measurement mode in the *m*/*z* range from 10 to 160 amu, with an accumulation time of 200 ms.

### 2.5 Calculation Methods

We used mainly nonperiodic (cluster) methods for geometry optimizations and frequency calculations of organic cations, water, and ozone molecules, as well as products found in the degradation process of these organic cations. All molecular optimizations were carried out using the conductor-like polarizable continuum model (CPCM) implicit solvent method,^46^ with water as the solvent. Initially, the tight binding method GFN2-xTB^47^ was used to model the reaction mechanism based on the nanoreactor procedure developed by Grimme et al.^48^ This has lower computational costs and allows systematic screening of many conformers and degradation products. Unfortunately, the implicit theoretical levels in the method did not model accurately the Criegee mechanism for the ozonolysis of alkenes,^49^ resulting in products with aromatic nitrogen heterocycles that were not compatible with the experimental results, in addition to CH_3_^+^, H_2_O_2_, and CO_2_, among other species. Consequently, we increased the theoretical level of the electronic structure calculation using the “Swiss army knife” *r*^2^SCAN-3c composite electronic structure method,^50^ which uses the *meta*-GGA *r*^2^SCAN exchange–correlation functional,^51^ a tailor-made triple-ζ Gaussian atomic orbital def2-mTZVPP basis, def2-mTZVPP/J auxiliary basis sets, and the density functional theory-D4 (DFT-D4) (version 2.5) dispersion model.^52^ This method can accurately calculate the van der Waals electronic dispersion energies between reactants and the interactions between the ozone molecule and the aromatic bonds of organic cations reproducing the first step of the Criegee mechanism with intermediate adduct formation in the process of organic cation degradation. We employed the ORCA code (version 5.0.3).^53^

Furthermore, determining the degradation mechanism requires calculation of the energy barriers between the reactant and product states (activation energies). To calculate these activation energies and locate the transition states, we utilized the nudged elastic band with transition state optimization method (NEB-TS), as implemented in the ORCA program. However, owing to its computational cost, we applied it only to cases of interest that could provide clues regarding the general aspects of the probable routes. Fukui functions were also calculated to study reactivity (see the Supporting Information (SI)).

## 3 Results and Discussion

### 3.1 Ozone Detemplation of HPM-16

#### 3.1.1 Infrared Spectroscopy

**Figure 1:**
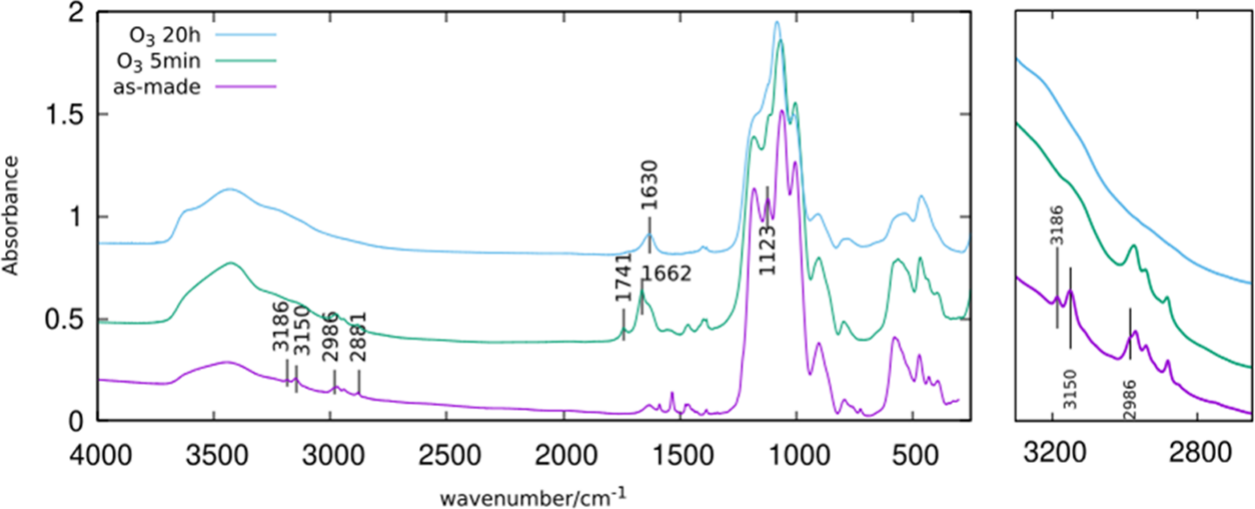
IR spectra in KBr of HPM-16: (from bottom to top) as-made and after 5 min and 20 h O_3_ treatment at 100 °C. At the right, the CH stretching region is shown enlarged.

Our most detailed investigation of the low-temperature detemplation process was done on the new large/medium-pore zeolite HPM-16, synthesized with 1M2E3nPrI. Figure 1 shows the FTIR spectra in KBr of HPM-16 in as-made form and after 5 min and 20 h of O_3_ treatment at 100 °C. The spectrum of as-made HPM-16 contains bands at 3150 and 3186 cm^–1^ assigned to the C–H stretching of the aromatic imidazolium ring as well as several overlapped bands in the 2840–3005 cm^–1^ range, assigned to the C–H stretching of the three aliphatic moieties of the OSDA. After just 5 min of O_3_ treatment, the aromatic bands totally disappeared, while the aliphatic ones remained mostly unaltered in both intensity and frequency. The most noticeable change in the aliphatic C–H stretching region concerns just a shoulder at 2986 of the 2970 cm^–1^ band in the as-made sample that cannot be detected in the broader and asymmetric 2975 cm^–1^ band of the 5 min treated sample. These changes suggest the first steps of the O_3_ detemplation are associated with a loss of aromaticity that does not significantly alter the aliphatic portions of the OSDA. After a 20 h treatment, no C–H stretching bands remain, suggesting the detemplation is complete. In addition to the C–H stretching region, changes after 5 min and 20 h treatment also occur in the O–H stretching region, with bands shifting in frequency and increasing in intensity, which is likely related to the increased water content, as supported by the increase in intensity of the water bending band at 1630 cm^–1^. Another band around 1400 cm^–1^, to be discussed below, also appears after the 20 h treatment.

The loss of aromaticity after 5 min treatment is also supported by several other facts, including the disappearance of the relatively sharp bands at 1587 and 1533 cm^–1^, assignable to the stretching of the imidazole ring. The appearance of bands at 1741 and 1662 cm^–1^ may suggest the formation of carbonyl groups by the oxidizing treatment with ozone. A sharp band at 1123 cm^–1^ in the spectrum of the as-made sample, which can be assigned to bending vibrations of the imidazolium ring,^54^ is also lost after the short ozone treatment.

**Figure 2:**
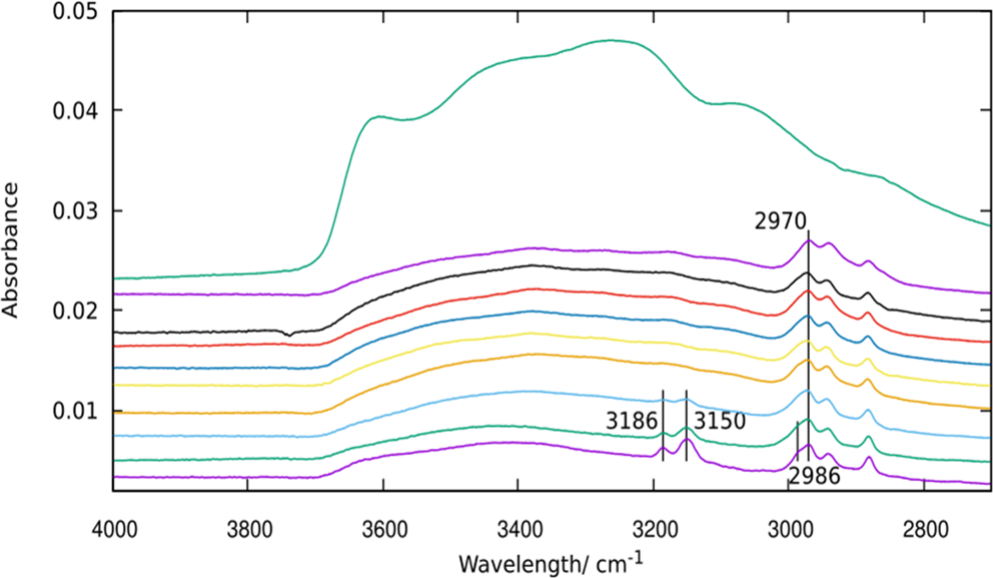
ATR-FTIR spectra in the X–H stretching regions of HPM-16 samples. From bottom to top: as-made and after O_3_ treatment at RT for 15 s and 5 min and at 100 °C for 30 s; 2, 5, and 20 min; and 1, 2, and 20 h.

**Figure 3:**
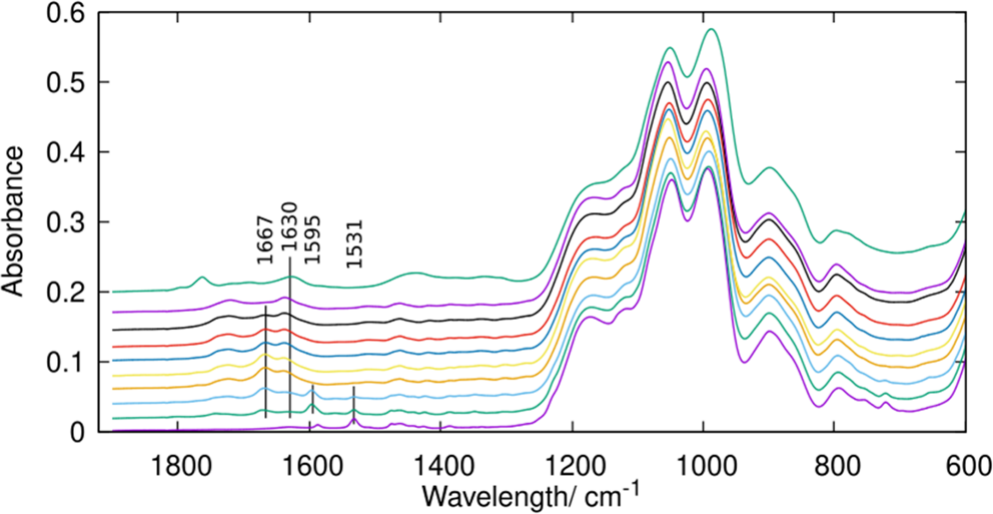
Low wavenumber region of ATR-FTIR spectra of HPM-16 samples. From bottom to top: as-made and after O_3_ treatment at RT for 15 s and 5 min and at 100 °C for 30 s; 2, 5, and 20 min; and 1, 2, and 20 h.

The kinetics of detemplation on HPM-16 has been followed in more detail by ATR-FTIR because of the easy and fast sample preparation with this technique. Despite the fact that the internal reflection element of the ATR accessory (a diamond crystal) absorbs all of the IR light in the 1900–2300 cm^–1^ region, according to the FTIR-KBr spectra shown in Figure 1, the obscured region does not seem to be relevant for this study. Spectra after treatment at room temperature and 100 °C were followed along time, as shown in Figures 2 and 3. The spectra show that even at room temperature, the O_3_ treatment is quite effective: the aromaticity after 15 s is already significantly reduced and almost totally disappears after just 5 min (Figure 2). The subsequent treatment at 100 °C eliminates the rest of the aromatic bands in just 30 s. By contrast, the aliphatic C–H stretching bands remain quite unaltered until a much longer treatment of 20 h at 100 °C totally removes them.

Again, the conclusions are supported by the spectra in the imidazole and carbonyl stretching regions, as bands at 1531 and 1586 (imidazole) disappear while bands at 1658 and 1667 cm^–1^ and in the 1720–1745 cm^–1^ region (carbonyl) appear after ozone treatment at room temperature (Figure 3). These later spectra also show a band at 1595 cm^–1^ that might tentatively be assigned to the N–O stretching of a nitro moiety, which disappears after only 30 s of treatment at 100 °C. These results suggest a potential route for nitrogen removal at 100 °C through the formation of an intermediate nitro compound. However, at least part of the two nitrogen atoms of the OSDA cation would be retained for longer times at this temperature, as will be discussed below. At the same time, the water bending band around 1630 cm^–1^ develops. These changes strongly suggest the aperture of the 5-atom imidazolium ring with the formation of carbonyl compounds. In the (almost) detemplated zeolite treated for 20 h, the most significant bands appear at 1625 (stretching of C=C or C=O, or N–H bending) and 1762 cm^–1^ (possibly C=O stretching in a carboxylic acid). Because of the relatively low concentration of organic species and the likely complex mixtures formed (see calculations below), it is difficult to make a complete assignment of all of the bands, but the first important conclusions are that the aromaticity readily vanishes upon ozonation while aliphatic rests remain longer and C=O groups develop. Because of the oxidizing conditions, the formation of carbonyl groups seems reasonable. Also, a broad band at ca. 1450 cm^–1^ appears in the spectrum of the sample treated with ozone at 100 °C for 20 h, which can be assigned to ammonium cations, as will be discussed below.

#### 3.1.2 MAS NMR Spectroscopy

**Figure 4:**
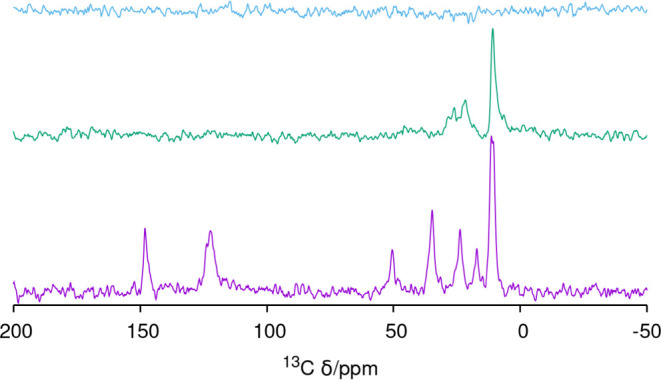
^13^C MAS NMR spectra of HPM-16 (from bottom): as-made^34^ and after 5 min and 20 h O_3_ treatment at 100 °C, showing that the aromaticity of the pristine OSDA (resonances around 122, 124, and 147 ppm) is lost very fast and a long treatment removes most of the template.

**Figure 5:**
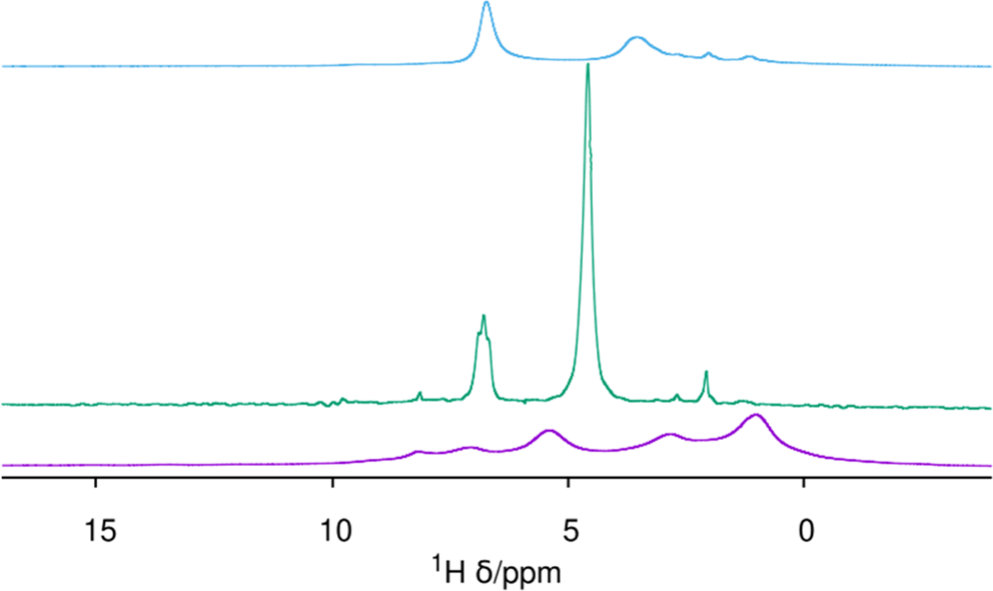
^1^H MAS NMR of HPM-16, from bottom: after 5 min and 20 h O_3_ treatment at 100 °C. The top spectrum is that of the 20 h treated sample after drying.

The ^13^C MAS NMR spectrum of the HPM-16 sample treated at 100 °C for 5 min agrees with the rapid loss of aromaticity (Figure 4). The spectrum shows resonances at around 10, 22, 26, and 28 ppm, all compatible with aliphatic C without bonds to charged N, and shows no resonances in the aromatic region. The ^1^H spectrum of the short-treated sample is quite complex and difficult to interpret, with at least 5 broad and overlapped resonances at around 8.1, 6.9, 5.3, 2.7, and 0.9 ppm (Figure 5). After 20 h treatment, no ^13^C signal is observed (Figure 4), and the ^1^H spectrum (Figure 5) is much simpler and presents sharper resonances at 4.6 ppm (main, assigned to water), 6.8 ppm, with shoulders at each side, and a much smaller one at around 2 ppm. We assign the 6.8 ppm signal to NH_4_^+^ in line with the observations made after ammonia adsorption on Brönsted acid sites in zeolites and silicoaluminophosphates.^55^ After drying this sample at around 100 °C overnight, the ^1^H MAS NMR spectrum changes significantly: the 4.6 ppm resonance disappears, in agreement with the above assignment, and two featureless resonances at 6.7 and 3.5 ppm develop. The 6.7 ppm resonance is again attributed to NH_4_^+^, while that at 3.5 ppm is assigned to dangling Si–OH groups in the interrupted **-HOS** framework.

**Figure 6:**
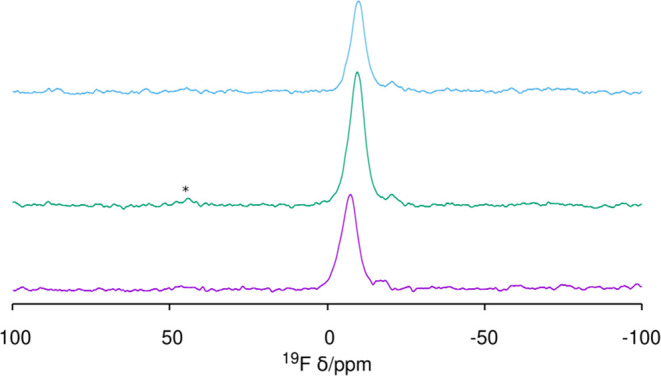
^19^F MAS NMR spectrum of HPM-16, from bottom: as-made^34^ and after 5 min and 20 h O_3_ treatment at 100 °C, demonstrating fluoride retention within *d4r* units during detemplation. A spinning sideband is marked with *.

In as-made HPM-16, there is one fluoride anion occluded in every *d4r* of the **-HOS** framework, as determined by crystallography.^34^ This is the most general case in zeolites prepared with fluoride and containing *d4r*.^19,28,33,45,56^ Most interestingly, the ^19^F MAS NMR spectrum in Figure 6 demonstrates F^–^ is still trapped in germanosilicate *d4r* units (F^–^@*d4r*) in HPM-16 after short and long O_3_ treatments, as deduced from a main resonance at around −10 ppm (type III, see ref (56) for assignments) and a very small one at −21 ppm (type II). Both spectra are notably similar to each other and also to the one recorded for the as-made zeolite in terms of approximate position and relative intensity.

#### 3.1.3 Mechanistic Calculations

We have investigated the mechanisms for the low-temperature O_3_-mediated degradation of the imidazolium OSDA in HPM-16 (1M2E3nPrI, 1-methyl-2-ethyl-3-*n*-propylimidazolium) using DFT calculations of product energies and transition structures, as well as through nudged elastic band with transition state optimization method (NEB-TS) calculations for activation energies (see the SI). In addition, we investigated other imidazolium cations relevant to this work (see below): Im (imidazolium), 123TMI (1,2,3-trimethylimidazolium), and 2E134TMI (2-ethyl-1,3,4-trimethylimidazolium), which allowed us to generalize the conclusions. Tekle-Röttering et al.^57^ described a degradation scheme for the neutral imidazole molecule with O_3_, where the heterocycle is attacked by the O_3_ molecule, forming a five-membered adduct between the aromatic C atoms (C4 and C5) of the neutral imidazole and O_3_ molecules, analogous to the molozonide adducts (or primary ozonides) formed as the first step in the ozonolysis of alkenes in the Criegee mechanism. Following the Criegee mechanism for the ozonolysis of unsaturated compounds,^58^ the C4–C5 bond is broken, resulting in formamide and formylisocyanate, followed by cyanate and formate. A similar mechanism has also been proposed for other imidazole-based molecules. For example, protonated histidine, which contains an imidazole group, is also degraded by O_3_ addition at low temperatures.^58^ Unfortunately, the literature does not clearly describe the reaction products. These products can be further degraded by hydrolysis or oxidation.^59^ For example, formamide can be hydrolyzed to ammonia and formic acid.^60^

In our system, and in contrast to previous studies, the imidazole cycle is not neutral but bears a positive charge, which is balanced by F^–^@*d4r*. Our experiments at low temperature (100 °C) show that the F^–^ anion remains trapped in the zeolite during the ozonolysis process, as seen in the ^19^F NMR results (see Figure 6 for HPM-16 and Figure S9 for other large-pore imidazolium-containing zeolites). A parsimonious hypothesis is that the F^–^ anion simply remains trapped in the *d4r* during the degradation of the organic cation and does not actively participate in the process. Removal of the F^–^ anion from the *d4r* does not likely involve the direct diffusion through a 4-membered ring, which would require a very high temperature.^61^ In a standard calcination, experiments definitely showed that the F^–^ anion escapes from the *d4r*, so Villaescusa et al. proposed that hydrolysis of Si–O–Si was required, although the *d4r* is completely restored after calcination.^62^ Zicovich-Wilson et al.^63^ studied this issue and calculated that the hydrolysis of one of the Si–O–Si bonds implies an energy barrier that is relatively high but accessible during calcination (∼52 kcal/mol). However, this mechanism is expected to be less accessible during ozonation because of the lower temperatures involved. The recent work by Wang et al. suggests cage aperture may occur at a temperature not much higher (175 °C).^37^

**Figure 7:**
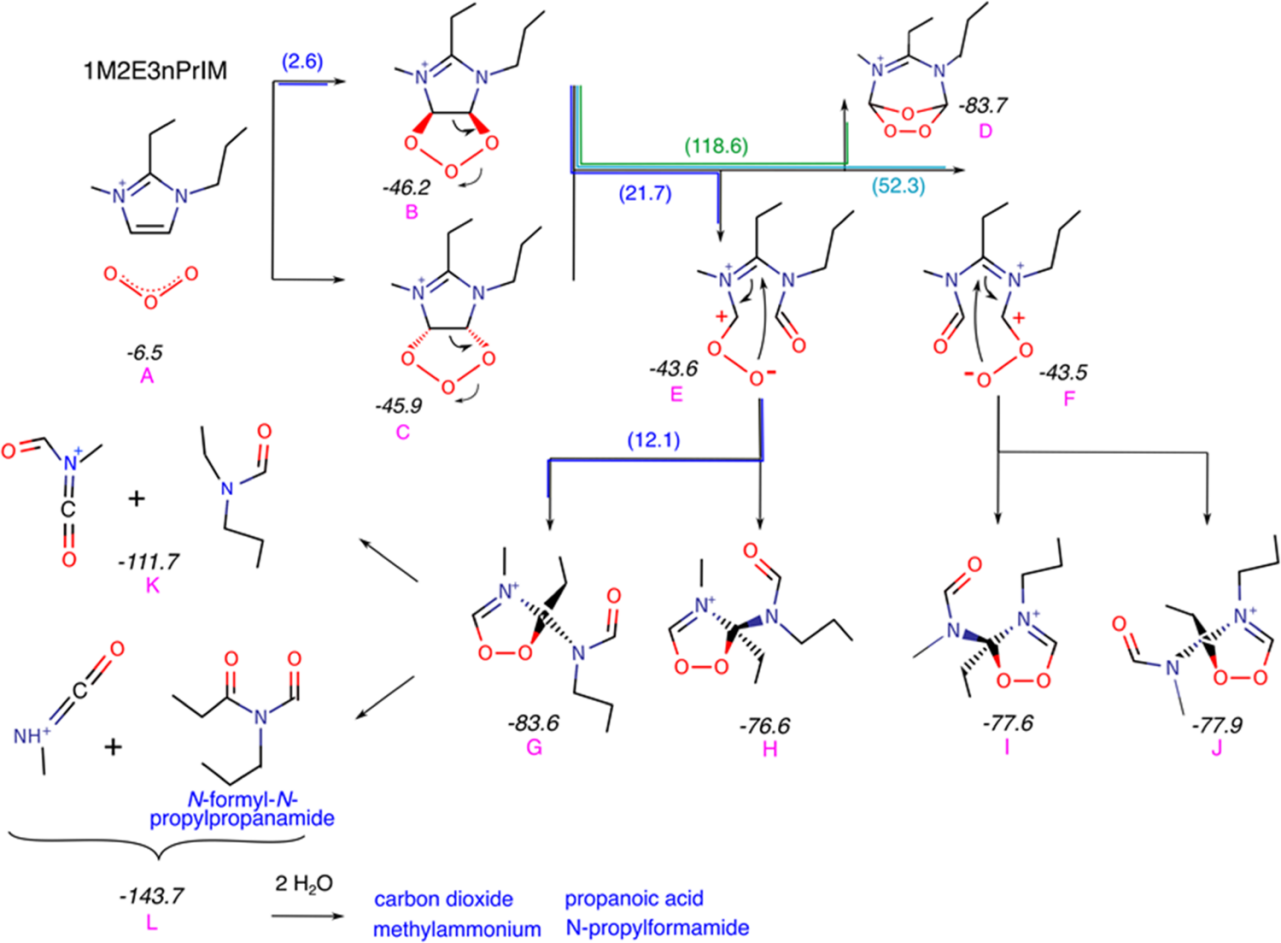
Reaction pathway (A → L, labels) proposed for the reaction of 1M2E3nPrIM with ozone in zeolites. Formation energies of each species in italic numbers (in kcal/mol). Some of the paths are marked with the activation energies (in parentheses). In blue text, we have highlighted the reaction products.

The general mechanism of degradation of the imidazole cations is depicted in Figure S1, which was obtained by studying the cations 123TMI, 2E134TMI, and 1M2E3nPrIM. We will now describe the mechanism of the ozonolysis of 1M2E3nPrIM cation more specifically (Figure 7). See Figure S2 for the other cations.

The cation 1M2E3nPrIM exhibits an attractive nonbonding interaction with O_3_, with a binding energy of −6.5 kcal/mol (Figure 7A). The union between the cation and O_3_ occurs when O_3_ attacks the C4=C5 double bond (Figure 7B,C), forming an adduct analogous to the molozonide formed with alkenes as a first step of the Criegee mechanism.^64^ As the 1M2E3nPrIM cation is not symmetrical with respect to the double bond, there is a slight energy difference between the different adducts formed. The energy of formation of the adduct relative to the isolated species is −46.2 kcal/mol with an activation energy of 2.6 kcal/mol. As a result of the reaction, the adduct (molozonide analog) loses its aromaticity, which explains the rapid disappearance of the bands at 3150 and 3186 cm^–1^ observed in the infrared experiments. This agrees with the calculated local reactivity, represented by the dual descriptor Δ (see Figure S3 for details). We have identified two main routes for adduct degradation: (a) ring opening and formation of multicharged cationic transition states (Figure 7E,F), with formation energies close to that of the adduct, and thermodynamically accessible activation energies (between 20 and 50 kcal/mol, depending on the locations of the charge densities of the N^+^ atom and –O_2_^–^ group), and (b) the formation of a Criegee ozonide (such as Figure 7D), which is reported in the literature in aprotic solvents,^59^ and which is the typical rearrangement of the molozonide in the ozonolysis of alkenes,^65^ but which, in our case, has a too high activation energy (118.6 kcal/mol) to be considered a viable degradation pathway. Thus, we have only considered the first main pathway. The multicharged cations E and F in Figure 7 quickly form more stable nonaromatic compounds in aqueous solution: we have identified several highly stable configurations (∼−80 kcal/mol) with activation energies of 12 kcal/mol. We include four of these configurations in Figure 7 with labels G, H, I, and J. These cationic species contain functional groups that are highly reactive for further hydrolysis and/or oxidation, so further degradation is expected, as stated by Lim et al.^59^ According to Fukui functions (see Figure S3), the molecule is highly reactive for nucleophilic and electrophilic attacks in the C–N and O–O bonds. We have focused on the cleavage of these bonds to continue exploring the degradation process. However, depending on the order and type of attack, many degradation products can be obtained. The calculated route with the lowest formation energy (−195 kcal/mol) was 1M2E3nPrIM + O_3_ + 2H_2_O → CO_2_ + methylammonium + propanoic acid + *N*-propylformamide. Other common degradation products were H_2_O_2_, ammonia, formamide, formic acid, acetic acid, and oxygen. In general, the free energy of the OSDA degradation would be ∼−190 and −200 kcal/mol.

At the end of the process, the countercation balancing the F^–^@*d4r* charge could be ammonium (which would agree with the lack of ^13^C NMR signals, Figure 4, and with the significant N content remaining in the zeolites treated at 100 °C, see Table 2) or some aliphatic versions (e.g., methylammonium), as suggested by calculations for the different cations studied (see the Supporting Information for details). According to calculations of the acid attack of the intermediate species of the degraded OSDAs, in some cases, it could even be H^+^ (see Figures S2–S3 and discussion in the SI). However, the N content is generally large enough to allow for NH_4_^+^ as charge balancing species when the temperature is not higher than 100 °C (please consider that the N-to-charge ratio is 2 in the imidazolium and only one in the ammonium cations). We will discuss this issue below. The fact that NH_4_^+^ is the final product under severe oxidizing conditions suggests it must be strongly stabilized by the interaction with F@*d4r* (see the SI).

**Table 2:** CHN Chemical Composition of Selected As-Made and O_3_-Treated Samples

zeolite		*T* (°C)^a^	time^b^	C (%)	H (%)	N (%)
SiO_2_-2E134TMI**-STW**	as-made		0	12.66	1.99	3.69
		200	72 h	0.92	0.27	0.38
(Ge,Si)O_2_-10BDMI-HPM-8	as-made		0	11.82	1.80	2.76
		100	20 h	4.14	1.08	1.58
(Ge,Si)O_2_-10BDMI-**POS**	as-made		0	11.22	1.71	2.62
		100	20 h	6.46	1.56	1.96
(Ge,Si)O_2_-1M3TMBI-HPM-14	as-made		0	11.15	1.57	2.71
		100	20 h	2.10	1.58	2.46
(Ge,Si)O_2_-1M2E3PrI-HPM-16	as-made		0	10.26	1.95	2.63
		100	5 min	7.29	1.89	2.40
		100	20 h	1.57	1.36	2.18
		150	50 h	0.28	0.84	0.16
(Ge,Si)O_2_-1M2E3PrI-**-SYT**	as-made		0	12.79	2.72	3.35
		100	6 h	9.50	2.40	3.04
		100	20 h	7.51	1.79	2.50
		100	30 h	2.15	1.30	2.94
SiO_2_-4BI-**MFI**	as-made		0	7.50	1.29	3.19
		150	24 h	0.82	0.52	0.60
SiO_2_-EABO-**ISV**	as-made		0	14.89	2.40	1.23
		180	84 h	3.62	0.66	0.32
SiO_2_-4bBnMI-beta	as-made		0	17.46	2.10	3.04
		100	20 h	5.68	0.97	2.07
SiO_2_-8bDMI-beta	as-made		0	11.80	1.97	2.78
		150	6 h	5.51	0.96	1.12

a Temperature.

b Time of the O_3_ treatment for the treated samples. Temperatures higher than 100 °C were only applied when long treatments at 100 °C did not afford significant detemplation.

#### 3.1.4 Temperature-Programmed Desorption Mass Spectrometry

**Figure 8:**
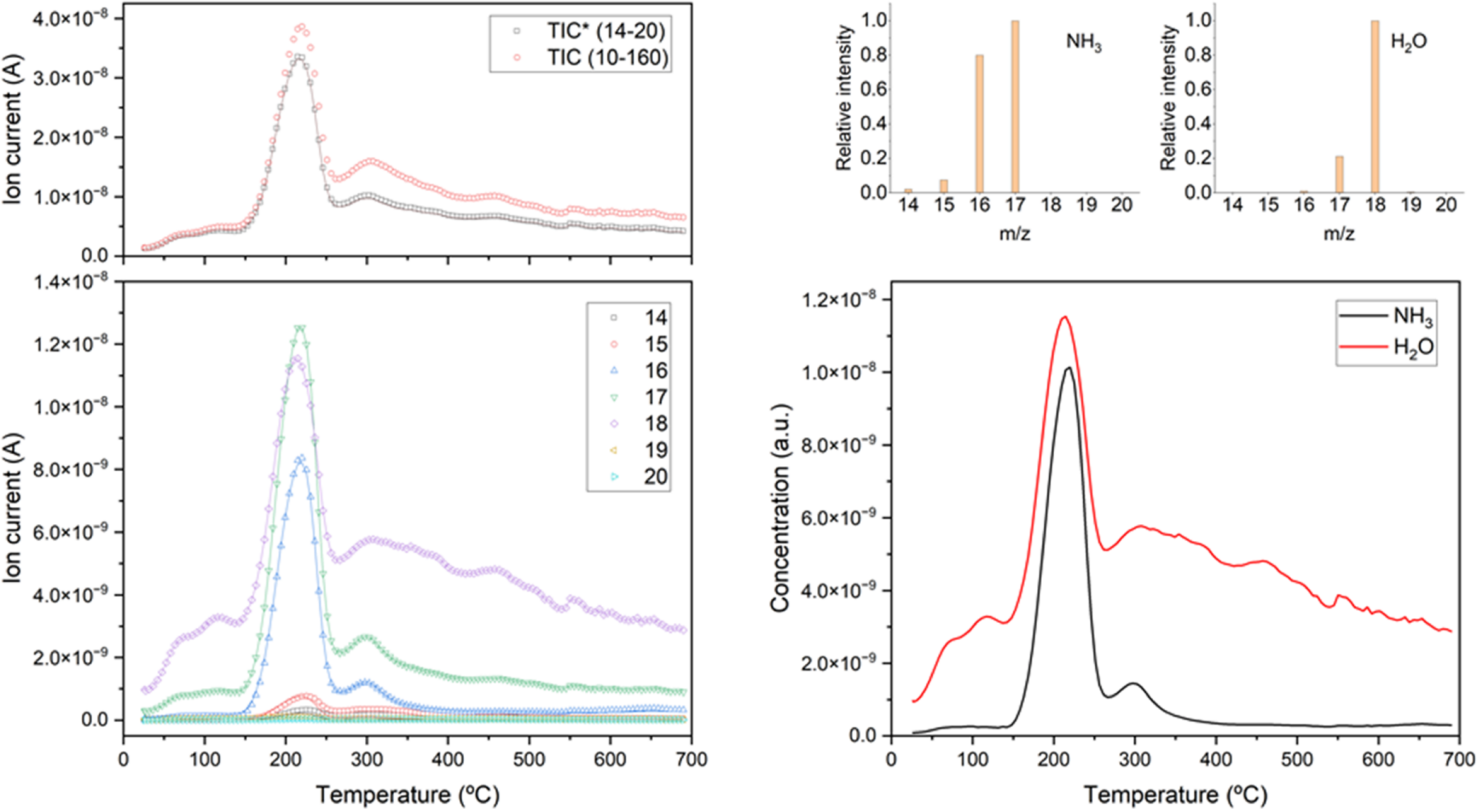
TPD-MS analysis of sample HPM-16 treated with ozone at 100 °C for 20 h. In the desorption curves at the left, the symbols correspond to measured ion current intensity for ions with *m*/*z* from 14 to 20 (bottom), and total ion current for ions with *m*/*z* from 14 to 20 (TIC*), and for the whole *m*/*z* range measured (TIC) (top), while lines indicate the corresponding fitted values. At the right, the reference mass spectra for ammonia and water used for fitting of ion current profiles (top) and calculated concentration profiles of desorbed water and ammonia (bottom) are displayed.

In order to get further insight into the type of species remaining in the sample after the ozone treatment, a temperature-programmed desorption analysis of the HPM-16 sample treated with ozone at 100 °C for 20 h was carried out. The sample was submitted to heating under dynamic vacuum from room temperature to 700 °C, and the evolved gases were analyzed by mass spectrometry (TPD-MS). The mass spectra showed signals corresponding to ions with mass-to-charge (*m*/*z*) ratios 16, 17, and 18 as the most intense, indicating that water and ammonia are the main products desorbed (Figure 8). Indeed, the intensity of all of the signals in the *m*/*z* range from 14 to 20 could be fitted as a combination of water and ammonia, using the electron ionization fragmentation patterns reported in the NIST database for these compounds.^66^ The calculated desorption profiles show a maximum desorption rate at temperatures around 200 °C for both water and ammonia. These results agree with the presence in the FTIR spectrum of this sample of bending vibration bands previously assigned to adsorbed water (1630 cm^–1^) and ammonium cations (1450 cm^–1^) (Figure 3), and also with the complex and intense bands that appear in the stretching region (Figure 2).^67^ This supports the presence of ammonium species in the sample treated with ozone at 100 °C for 20 h, which would decompose upon thermal treatment releasing ammonia. We noted, however, that the 1450 cm^–1^ band is not evident at all in the FTIR spectrum of the same treated sample taken by the KBr pellet technique (Figure 1, top). This is very likely due to the solid-state exchange of NH_4_^+^ by K^+^ during the preparation of the pellet, a phenomenon that must be taken into account when using this technique.^68^ In fact, the KBr spectrum in Figure 1, top, shows, instead of the band at 1450 cm^–1^, another one at 1400 cm^–1^, which can be assigned to NH_4_Br.^69^ In full agreement with our TPD-MS and ATR results and conclusions, a TPD-FTIR study of the HPM-16 sample after treatment at 100 °C for 20 h, in transmission mode, using a self-supported pellet, shows the existence of both water and ammonium in the original ozonated sample and its gradual removal as the temperature increases from 80 up to around 200 °C under vacuum (Figure S4).

It should be noted that, as this ammonium species would charge balance the fluoride anions, it could be expected that the removal of ammonium species would be accompanied by desorption of HF. Indeed, the mass spectra show very weak signals of fragments with *m*/*z* values 19 and 20, in addition to those corresponding to water, which might be due to the desorption of HF. However, these signals are extremely weak and cannot be unambiguously attributed to the desorption of HF. Nevertheless, it is very likely that if HF were released, it would react with the quartz reactor, thus preventing this compound being detected.

### 3.2 Ozone Detemplation on Other Zeolites

For other imidazolium-containing zeolites with relatively large pores (HPM-7, HPM-8, Figures S5–S6, respectively) detemplation by O_3_ was also achieved at a relatively low temperature (100 °C) after 20 h, as demonstrated by the (almost total) disappearance of the C–H stretching bands (both aromatic, 3100–3200 cm^–1^, and aliphatic, 2800–3000 cm^–1^), the presence of bending bands assignable to H_2_O (1630 cm^–1^) and NH_4_^+^ (around 1450 cm^–1^), and the large increase of broad and overlapped bands (2800–3700 cm^–1^) in the O–H and N–H stretching region. Bands in the 1720–1760 cm^–1^ range also develop, suggesting the presence of carbonyl groups in the final materials, as was also the case for O_3_-treated HPM-16. After treatments at 100 °C, significant amounts of N (and sometimes C) always remain (Table 2), again suggesting ammonium species, with or without alkyl groups, are charge balancing at least a portion of F^–^ anions. In the case of HPM-14, which has elongated extra-large 16-ring pores running only in one direction, the treatment significantly reduces but does not totally eliminate the bands in the imidazole ring-stretching region (Figure S7B) and some bands in the aromatic CH stretching region might also remain (Figure S7A), reflecting limitations to O_3_ diffusion. The conclusions drawn from infrared spectroscopy are validated also by ^13^C MAS NMR spectroscopy, which shows that while treated HPM-7 and HPM-8 show hardly any detectable resonances (only in the aliphatic region), treated HPM-14 still shows significant resonances in both aromatic and aliphatic regions (Figure S8). On the other hand, ^19^F MAS NMR shows that also in these three zeolites, F^–^ is retained in the *d4r* cages after the treatment (Figure S9).

With regard to zeolite **-SYT**, this contains both extra-large pores and an independent system of small 8-ring pores and, in this case, a relatively long treatment at 100 °C for 6 h afforded the almost complete disappearance of aromatic and partly aliphatic bands, with the possible formation of C=O (Figure S10). After a longer treatment of 20 h, there were almost no aliphatic bands in the ATR-FTIR spectrum. Again, after treatments at 100 °C, significant amounts of N still remain in the samples (Table 2). The fact that **-SYT** with nanocrystal size and both extra-large and small pores is harder to detemplate than **-HOS**, with larger crystal size (Table 1) but only large and medium pores suggests that pore size is more relevant for ozone-driven detemplation than crystal size.

**Figure 9:**
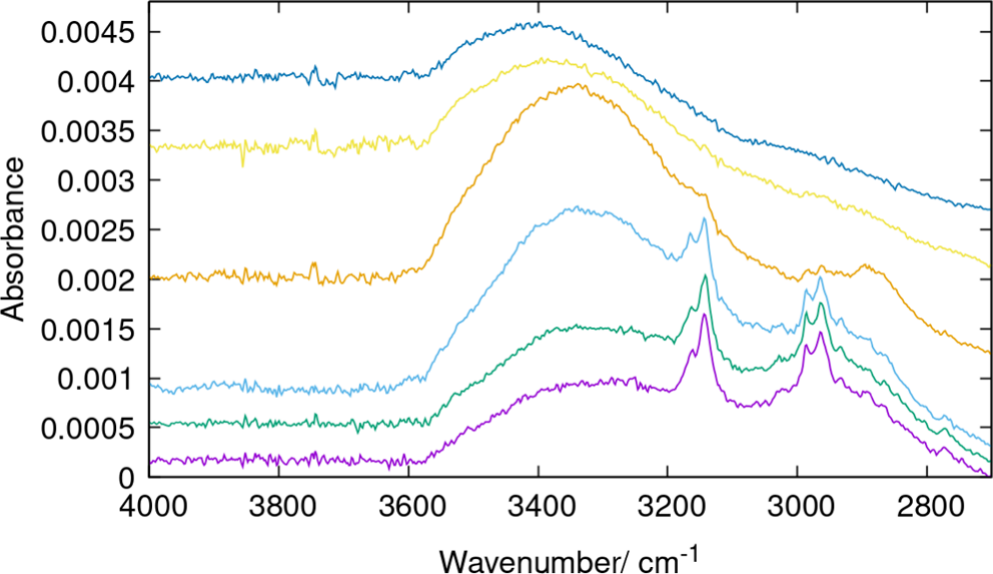
ATR infrared spectra in the C–H stretching region of pure silica **STW** zeolite synthesized using 2E134TMI (from bottom): as-made and after treatments in O_3_ at 100 °C (20 h), 150 °C (20 h), 200 °C (24 h), 200 °C (48 h), and 200 °C (72 h).

By contrast, much harder conditions were needed for the detemplation of the pure silica HPM-1 (**STW**). Contrary to the rest of the samples, **STW** has only medium and small pores, so diffusion problems are not unexpected. Also, the formation of the adduct between O_3_ and the imidazolium ring may be sterically hindered inside the *stw* cage. For **STW**, a 20 h treatment at 100 °C, even followed by 20 h at 150 °C had little impact on the aromatic and aliphatic C–H stretching regions. After a treatment at 200 °C for 24 h, these stretching bands almost totally disappeared (Figure 9), and the zeolites contained little N (Table 2).

**Figure 10:**
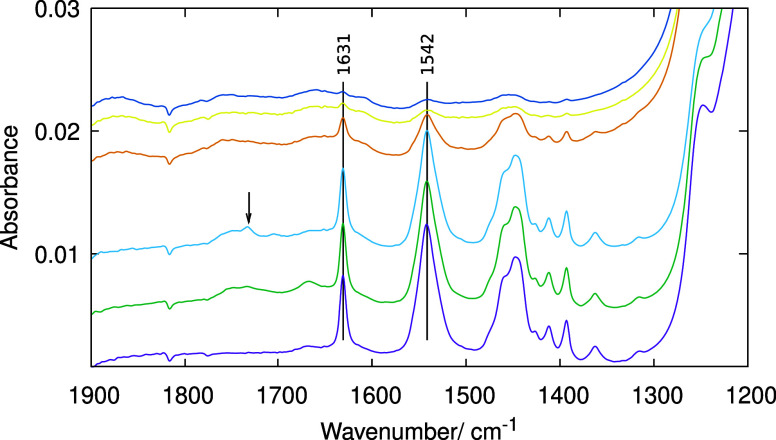
ATR infrared spectra in the imidazole ring-stretching region of pure silica **STW** zeolite synthesized using 2E134TMI (from bottom): as-made and after treatments in O_3_ at 100 °C (20 h), 150 °C (20 h), 200 °C (24 h), 200 °C (48 h), and 200 °C (72 h). The arrow points to a weak band assignable to carbonyl groups that only appears in the sample treated at 150 °C (20 h).

An analysis of the imidazole ring-stretching region in Figure 10 indicates the presence in treated **STW** of bands corresponding to C=N stretching (1631 cm^–1^), antisymmetric C=N–C=C stretching (1542 cm^–1^), and symmetric C=N–C=C (around 1450 cm^–1^),^70^ which shows that these bands are unaffected by 100 °C treatments, little affected at 150 °C, and much reduced (but not completely eliminated) by long treatments at 200 °C. The spectra do not show clear evidence for carbonyl compounds except for a small band at 1732 cm^–1^ in the sample treated at 150 °C for 2 h. Possibly, strong diffusion limitations hinder the formation of large amounts of the corresponding compounds.

In the framework vibration region, Figure S11, the spectra of the sample after treatment at 100 or 150 °C are much similar to each other and clearly different from those treated at 200 °C, supporting that most of the organic matter is removed only after treatment at 200 °C.

**Figure 11:**
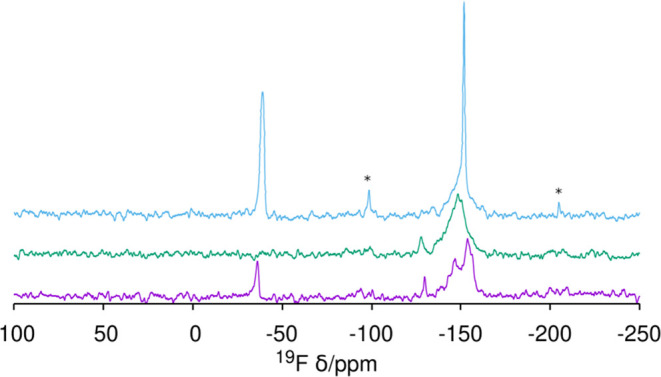
^19^F MAS NMR of pure silica zeolites treated with O_3_ at the *T* and time indicated: (from bottom to top) **STW** 200 °C (72 h), **MFI** 150 °C (24 h), and **ISV** 180 °C (84 h). Spinning side bands are marked with *.

**Figure 12:**
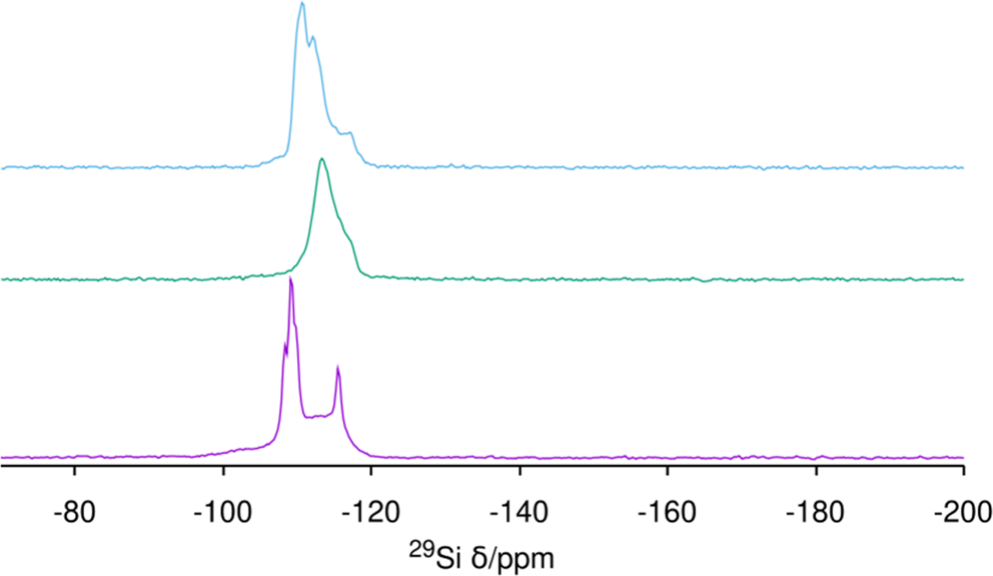
^29^Si MAS NMR of pure silica zeolites treated with O_3_ at the *T* and time indicated: (from bottom to top) **STW** 200 °C (72 h), **MFI** 150 °C (24 h), and **ISV** 180 °C (84 h).

The ^19^F MAS NMR spectrum after the 200 °C treatment of **STW** shows that in that case, fluoride is not retained in the *d4r* cages since the most relevant resonances occur around −150 ppm and the signal at −36 ppm, characteristic of F^–^@*d4r* in pure silica **STW**, is only around 10–12% of the total signal (Figure 11, bottom). In our opinion, this residual F^–^@*d4r* may be due to the hampered diffusion of O_3_ in this medium/small-pore zeolite leaving portions of the crystals unaffected. The ^29^Si MAS NMR spectrum of the treated **STW** sample is also informative due to the absence of Ge in the framework. As shown in Figure 12 bottom, the spectrum is much different from the as-made **STW** (two distinct resonances at −106 and −113 ppm)^18^ and resembles more the calcined material (four well-resolved resonances in the −107 to −115 ppm range)^17^ but with a lower resolution and with the clear presence of *Q*^3^ defect sites. The percentage of *Q*^3^ suggests less than half of the *d4r* cages are broken (*Q*^3^ < 10% Si, compared to 20% if every *d4r* were broken with a SiOH HOSi pair). This indicates that at these treatment temperatures, the *d4r* cage in **STW** can be opened to allow F out, but it is not so easily reconstructed, as it occurs in a standard high-temperature calcination.^62^ The difference between SiO_2_**STW** and SiO_2_**ISV** (which at 180 °C shows no defects even if around 2/3 of F has been removed, see below) might be due to the known high rigidity of the SiO_2_**STW** framework.^71^ Since the ^19^F MAS NMR in Figure 11, bottom, shows resonances at high field that cannot be related to any ^29^Si resonances in Figure 12 corresponding to a coordination higher than four, we assign those resonances to organic fluorides, possibly including organoammonium fluorides.^72^

We have next considered two additional types of pure silica zeolites: a medium-pore **MFI** synthesized with an imidazolium cation but lacking the *d4r* units and a large-pore **ISV** containing *d4r* but synthesized with a quaternary ammonium cation. For both zeolites, the ozonolysis proved difficult. After O_3_ treatment at 100 °C for 24 h, the OSDA in **MFI** zeolite still showed the aromatic C–H bands in the 3000–3200 cm^–1^ region almost intact. The aromaticity could be eliminated after a 24 h treatment at 150 °C. However, ^19^F MAS NMR showed resonances only in the −150 ppm region (Figure 11, middle), while the pristine zeolite had them at −68 and −79 ppm.^39^ This suggests fluoride is not retained in this case inside the 4^1^5^2^6^2^ cage of **MFI**, which may be due to the higher-temperature treatment and/or the larger windows of that cage. Defects in the final material are not evident in this case by ^29^Si MAS NMR (Figure 12, middle).

In the **ISV** case, which contains a quaternary ammonium spiro bicycle instead of an imidazolium, the detemplation proved even more difficult despite the much larger pores of **ISV** compared to **MFI**, HPM-16, HPM-14, or HPM-7. 24 h O_3_ treatments at 100 and 150 °C had almost no impact in the C–H stretching region of the infrared spectra, indicating lower reactivity of the alkylammonium cation with ozone. Extended treatments at 180 °C were necessary to significantly decrease the C–H stretching bands in this case. The ^19^F MAS NMR spectrum proves most fluoride anions have left the *d4r* cage, but around one-third remain in the cavity, according to the relative intensity of the corresponding resonance (around −39 ppm, type I, i.e., F in SiO_2_-*d4r*).^62^ This, however, most likely reflects the incomplete removal of the OSDA, which is suggested by the C/N and H/N ratios in the zeolite (13.2 and 28.7, respectively see Table 2) which are close to the ones in the pristine cation (14 and 26, respectively).^13^C MAS NMR leaves the issue clear since all resonances correspond to the OSDA occluded in the zeolite (Figure S12). The residual amount of N roughly agrees with a one-third of OSDA remaining. As a conclusion, quaternary ammonium cations appear to be much more resistant to the ozonolysis treatment and require conditions too harsh to maintain the fluoride anion in the *d4r* cage. *Q*^3^ resonances are not obvious in the final ^29^Si MAS NMR spectrum (Figure 12, top). Hence, fluoride removal from *d4r* in **ISV** with cage reconstruction at a temperature of 180 °C agrees with the findings in Wang’s work.^37^

**Figure 13:**
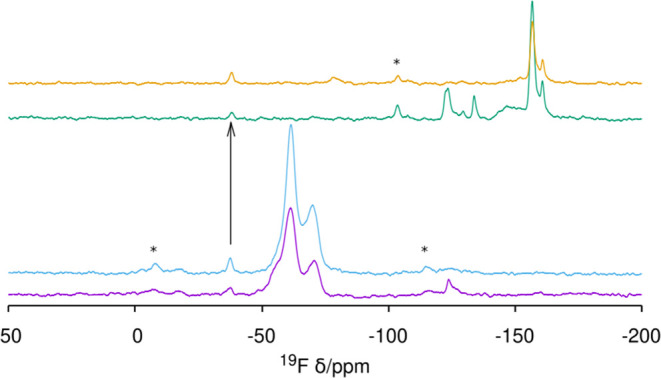
^19^F MAS NMR of pure SiO_2_ beta zeolites (from bottom): as-made 4bBnMI-beta and 8bDMI-beta and after O_3_ treatment of 4bBnMI-β at 100 °C (20 h), and of 8bDMI at 100 °C (20 h) followed by 150 °C (6 h). The arrow points to the resonance corresponding to F^–^@*d4r*, which is retained after treatment. Spinning side bands are marked with *.

The observations presented in this work demonstrate that imidazolium OSDAs may be removed relatively easily from silicogermanate zeolites with large pores and that this results in fluoride retention in *d4r* cages. There is, however, one question remaining: is Ge necessary for either F retention or easy imidazolium removal? To answer that question, we need to test a large-pore pure silica zeolite with fluoride in *d4r* prepared through the use of an imidazolium OSDA. We have used SiO_2_ beta prepared with imidazolium because, as it usually happens by the fluoride route, some polymorphs containing *d4r* exist in the beta intergrowth.^73^ We first tested the existence of F^–^@*d4r* in two as-made SiO_2_ beta samples prepared by the fluoride route using imidazolium cations. As shown in Figure 13, both zeolites do contain F^–^@*d4r* (−37 to −38 ppm). Ozone treatment at 100 °C for 20 h sufficed to remove the OSDA in the sample synthesized with 4bBnMI, while the sample prepared with 8bDMI required an additional 6 h treatment at 150 °C (Figures S13 and S14). Importantly, the ^19^F spectra show that the fluoride anions occluded in SiO_2_*d4r* are retained, while those in other cages (−50 to −75 ppm) are not (Figure 13). Fluoride retention in *d4r* in our work at T no higher than 150 °C sharply contrasts with the results by Wang et al. at 175 °C,^37^ where fluoride was removed from *d4r*, although it was retained somewhere else in the zeolite. F removal from *d4r* in that recent work and our own results for **ISV** at 180 °C suggest that there is a critical temperature in the narrow 150–175 °C range, above which fluoride can leave the *d4r* cage.

### 3.3 Degermanation and Stability Tests

**Figure 14:**
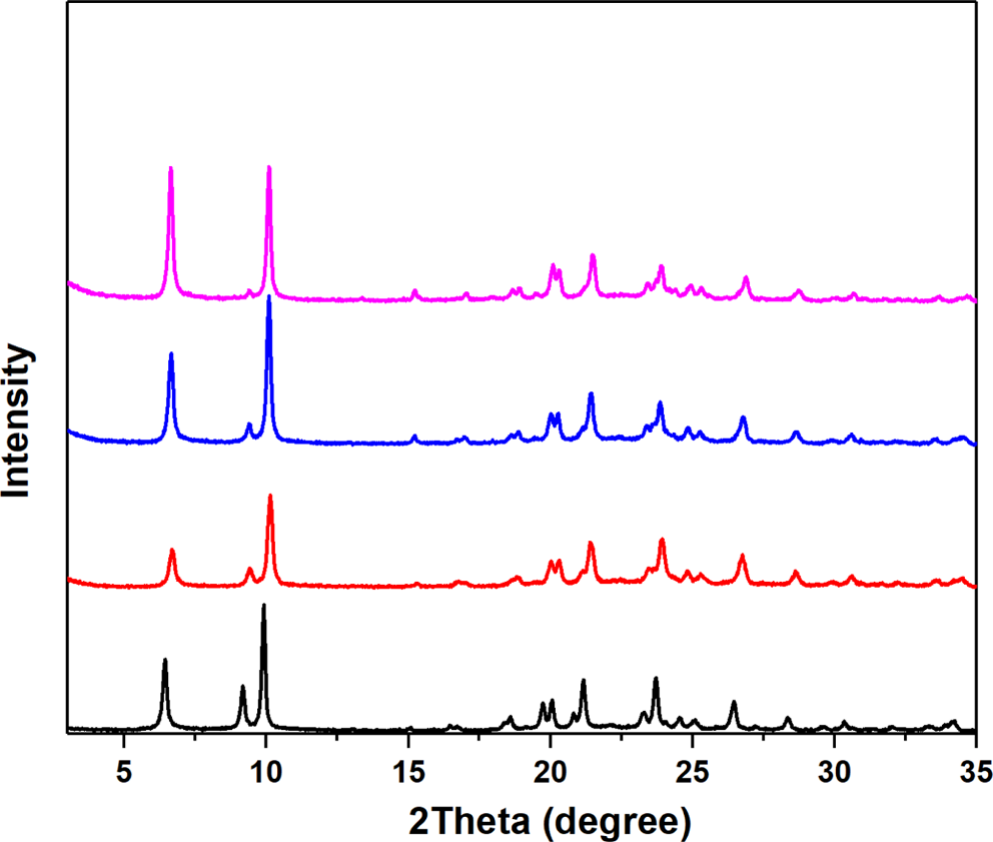
PXRD patterns of HPM-7: (from bottom to top) as-synthesized, after ozone treatment at 100 °C, after first run of degermanation, and after last run of degermanation.

**Figure 15:**
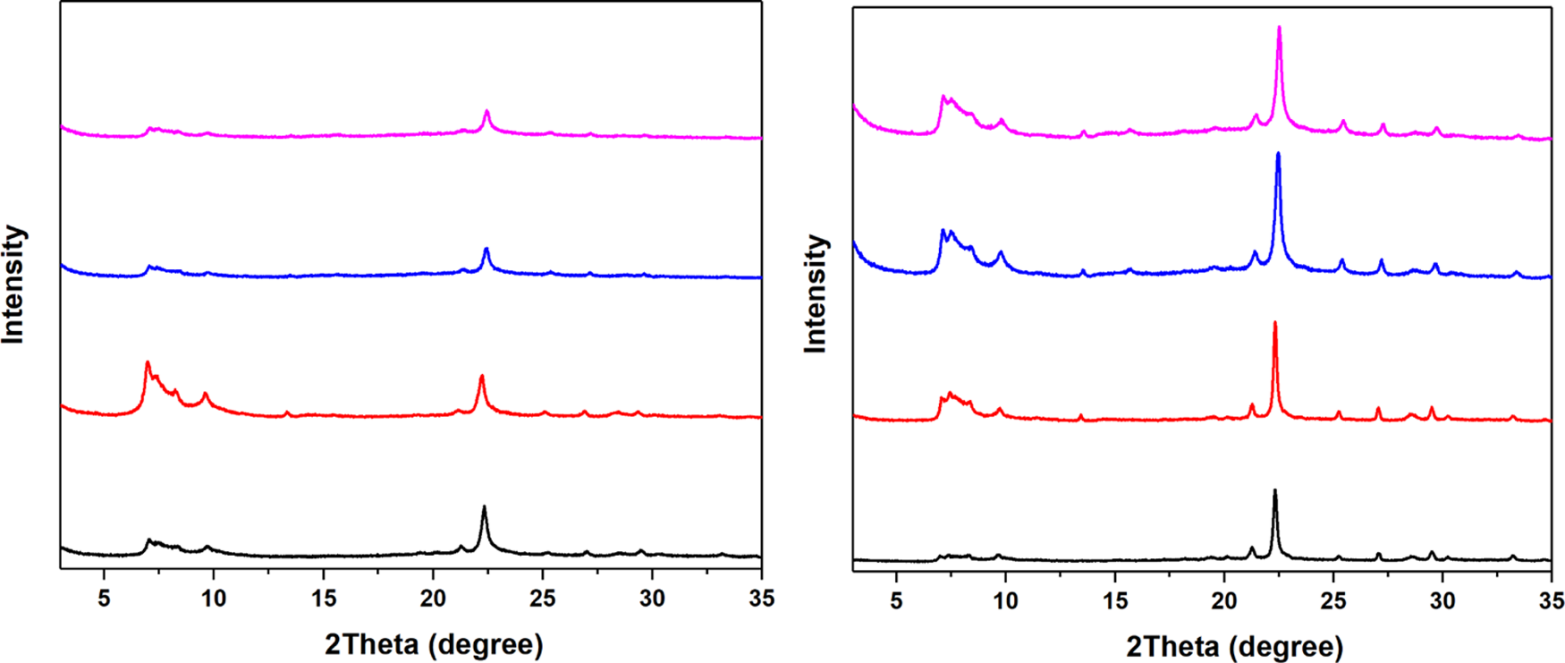
PXRD patterns of HPM-8 detemplated in air at 500 °C (left) and in ozone at 100 °C (right); from bottom to top: as-synthesized, detemplated (air calcination or ozone treatment), after first run of degermanation, and after last run of degermanation. Both left and right figures span the same intensity range, and patterns are vertically offset by the same amount. All of the patterns were collected on the same sample holder with similar amount in order to make the intensities comparable.

In several germanosilicate zeolites, it is possible to use the low-temperature ozone-treated zeolite to remove Ge and get a stable zeolite (Table 3). This occurs in the case of HPM-7, HPM-8, and HPM-16 but not in the case of HPM-14 and SYSU-3, which contain a larger Ge content and larger pore sizes. After the ozone treatment and degermanation, HPM-14 converts to an amorphous phase (Figure S15), which could be explained by the very high Ge_f_ of the initial material. For the zeolites with Ge_f_ = 0.3 in the as-made form, HPM-7 and HPM-16, a treatment with ozone at 100 °C afforded the subsequent degermanation using a mild acidic hydrothermal treatment with HCl-EtOH 0.75% solution (Figure 14 and ref^34^, respectively). However, under similarly mild degermanation conditions, the 24-MR extra-large-pore zeolite SYSU-3 collapses (Figure S16), possibly caused by the very high concentration of *d4r* in the framework. As for the HPM-8 possessing the lowest Ge_f_, a conventional calcination at 500 °C and an ozone-treatement at 100 °C were compared, and the ozone-treated sample shows better stability and crystallinity after degermanation using 1 M HCl aq (Figure 15). The quasi-pure-silica nature of the resulting HPM-8 was confirmed by EDS, and its ^29^Si MAS NMR spectrum indicates a predominance of *Q*^4^ Si sites at −111.8 and −114.5 ppm, and small amount of *Q*^3^ sites at −102.0 and −105.0 ppm, which could be due to defects arising during the acid treatment. All of these results prove that milder ozone detemplation can be used as a method to stabilize the germanosilicate zeolites, compared with the high-temperature calcination.

**Table 3:** Degermanation Details and Results on the Zeolites Studied in This Work

zeolites	detemplation method	degermanation method	Ge_f_ initial	Ge_f_ first deger	Ge_f_ final deger	stabilities
HPM-16	500 °C air for 6 h	0.75% HCl-EtOH 120 °C for 1 day	0.30	n.a.	n.a.	unstable
HPM-16	100 °C O_3_ for 20 h	0.75% HCl-EtOH 120 °C for 1 day	0.30	0.26	0.09	800 °C thermal stable
HPM-8	550 °C air for 6 h	1 M HCl (aq) 120 °C for 1 day	0.15	n.a.	n.a.	poor crystallinity
HPM-8	100 °C O_3_ for 20 h	1 M HCl (aq) 120 °C for 1 day	0.13	0.04	<0.01	hydrothermal stable in 1 M HCl at 120 °C
HPM-7	100 °C O_3_ for 20 h	0.75% HCl-EtOH 120 °C for 1 day	0.26	0.18	0.09	stable
HPM-14	100 °C O_3_ for 20 h	0.75% HCl-EtOH 120 °C for 1 day	0.69	n.a.	n.a.	unstable
SYSU-3	100 °C O_3_ for 6 h	0.75% HCl-EtOH 120 °C for 1 day	0.30	n.a.	n.a.	unstable

## 4 Conclusions

Removal of OSDAs from zeolites by O_3_ treatment at low temperature (100 °C) is generally feasible when the OSDA is based on the imidazolium ring and the pore system is large enough in all channel directions. The first steps of the process are rather fast, with the formation of a five-ring adduct between O_3_ and carbons 4 and 5 of the imidazolium ring, resulting in the loss of aromaticity of the OSDA as deduced from DFT calculations and in agreement with infrared spectroscopy results. Fluoride occluded in *d4r* of the zeolites is retained in the process if the temperature is not above 150 °C, and its charge is balanced by NH_4_^+^ after OSDA removal. For zeolites with medium pores, a higher temperature is required for total detemplation and F is not retained neither in the *d4r* (**STW**, 200 °C) nor in larger cages (**MFI**, 150 °C). Quaternary ammonium cations are considerably more resistant to the ozonolysis, so full detemplation of pure silica **ISV** could not be achieved even after a long treatment at a relatively high temperature (180 °C, 84 h). That treatment results in fluoride leaving the *d4r* cage (except for the fraction that presumably is still balanced by the quaternary ammonium cation). Experiments with pure silica beta zeolites synthesized using imidazolium OSDAs and containing a small fraction of F^–^@*d4r* shows, however, that F^–^@*d4r* is stable, even at 150 °C. Unfortunately, no pure silica zeolite with large pores synthesized with imidazolium and containing all F in *d4r* is known so far, but our results suggest such zeolite could afford a SiO_2_ zeolite with acidic properties of interest in catalysis.

## Supporting Information Available

Fukui functions and general mechanism for OSDA degradation (Figures S1–S3), and additional spectra (FTIR, ATR-FTIR, ^13^C, ^19^F, ^29^Si MAS NMR) and PXRD patterns (Figures S4–S17) (PDF)
