# Supplementary material for: Mechanism of the Low-Temperature Organic Removal from Imidazolium-Containing Zeolites by Ozone Treatment: Fluoride Retention in Double-4-Rings
Source: Inorg Chem. 2024 May 17;63(21):9953–66. doi: 10.1021/acs.inorgchem.4c01021 (PMC11134512; doi:10.1021/acs.inorgchem.4c01021)
Supplement: Supplementary file 1 — ic4c01021_si_001.pdf [file ic4c01021_si_001.pdf]

Mechanism of the Low Temperature Organic Removal from Imidazolium-Containing Zeolites by Ozone Treatment. Fluoride Retention in Double-4-Rings

Zihao Rei Gao,[a] Carlos Márquez Álvarez,[b] Salvador R. G. Balestra,[a,c] Huajian Yu,[a] Luis A. Villaescusa,[d] Miguel A. Camblor[a,*]

[a]Instituto de Ciencia de Materiales de Madrid (ICMM), CSIC, c/ Sor Juana Inés de la Cruz 3, 28049 Madrid, Spain.

[b] Instituto de Catálisis y Petroleoquímica (ICP), CSIC, c/ Marie Curie 2, 28049 Madrid, Spain.

[c] Departamento de Sistemas Físicos, Químicos y Naturales, Universidad Pablo de Olavide, Ctra. Utrera km 1, ES-41013 Seville, Spain.

[d] Instituto Interuniversitario de Investigación de Reconocimiento Molecular y Desarrollo Tecnológico (IDM) Universitat de València–Universitat Politècnica de València, Camino de Vera s/n, 46022, Valencia, Spain; CIBER de Bioingeniería Biomateriales y Nanomedicina (CIBER-BBN), Spain; Departamento de Química, Universitat Politècnica de València, Camí de Vera s/n, 46022, Valencia, Spain.

*Corresponding author: macamblor@icmm.csic.es

Supplementary information

S1. Fukui functions

Fukui functions were also calculated to study reactivity (see **Fig. S3**). We have used the nucleophilic and electrophilic Fukui functions f^+, f^-, and f^0 as defined by Parr and Yang,[1] and the dual descriptor Δf as defined by Morell *et al.*[2,3] to study the local reactivity by analyzing the electron density of the DFT calculations at r^2SCAN-3c level. The dual descriptor is an accurate function to

describe the local reactivity, as studied by Martínez-Araya *et al.*[4] We have calculated the Fukui functions from the electronic density of the molecules with *N*, *N+1*, and *N-1* electrons:

$$
\begin{aligned}
f^+(\vec{r}) &= \rho_{N+1}(\vec{r}) - \rho_N(\vec{r}), \\
f^-(\vec{r}) &= \rho_N(\vec{r}) - \rho_{N-1}(\vec{r}), \\
f^0(\vec{r}) &= \frac{1}{2}\left[\rho_{N+1}(\vec{r}) - \rho_{N-1}(\vec{r})\right], \\
\Delta f(\vec{r}) &= f^+(\vec{r}) - f^-(\vec{r}) = \rho_{N+1}(\vec{r}) - 2\rho_N(\vec{r}) + \rho_{N-1}(\vec{r})
\end{aligned}
$$

where f^+, and f^- describe the ability of an atom to lose an electron (nucleophilic attack) or to gain an extra electron (electrophilic attack), and f^0 denotes the neutral reactivity (radical attack). Therefore, molecular sites with $\Delta f > 0$ are expected to be susceptible for an electrophilic attack, whereas molecular centers with $\Delta f < 0$ are expected to be susceptible for a nucleophilic attack.

S2. General mechanism

The general mechanism is displayed in **Fig. S1**. The activation energy of adduct B is accessible at low temperatures (~ 3 kcal mol^{-1}, depending on the C4 and C5 branches); therefore, process A → B is highly probable. The rearrangement of this adduct to product C passes through a high activation barrier (> 50 kcal mol^{-1}); therefore, we consider the B → D pathways to be dominant (but strongly dependent on the branches, with a range of 20 – 50 kcal mol^{-1} activation energies). These latter pathways can generate multiple conformers (multicharged cation in case D), depending on the composition of the R1 – R5 branches, in three main routes, as highlighted in **Fig. S1**. Routes D → E and D → F led to heterocyclic peroxide cations. Route D → G requires a water molecule, which generates hydrogen peroxide. The final intermediates (E, F, and G) are highly reactive to molecules with -OH groups, which can be produced during the initial phases of ozone treatment from the interrupted -SiOH groups of the structure (internal or from the surface), and they can be available as reactants in the zeolite pore along with O_3, H_2O_2 (produced in the D → G pathway), and remaining zeolitic water. For the sake of simplicity, we assume that the degradation process of cations occurs in the presence of water and ozone, so successive cycles of degradation through hydrolysis and oxidation were studied to find clues about the possible final products. Successive degradation steps were studied sequentially for Im, 123TMI, 2E134TMI, and 1M2E3nPrIM (see the **Fig. S2** and **Fig. 7** in the main text). Although there are no samples with the Im cation, it has been used to rationalize in a more general way (see **Fig. S1**) the imidazolium ring cleavage. The attack sites focused on the cleavage of the C-N bonds (with activation free energies of 10-20 kcal mol^{-1}), as suggested by the Fukui functions calculations (see **Fig. S3**).

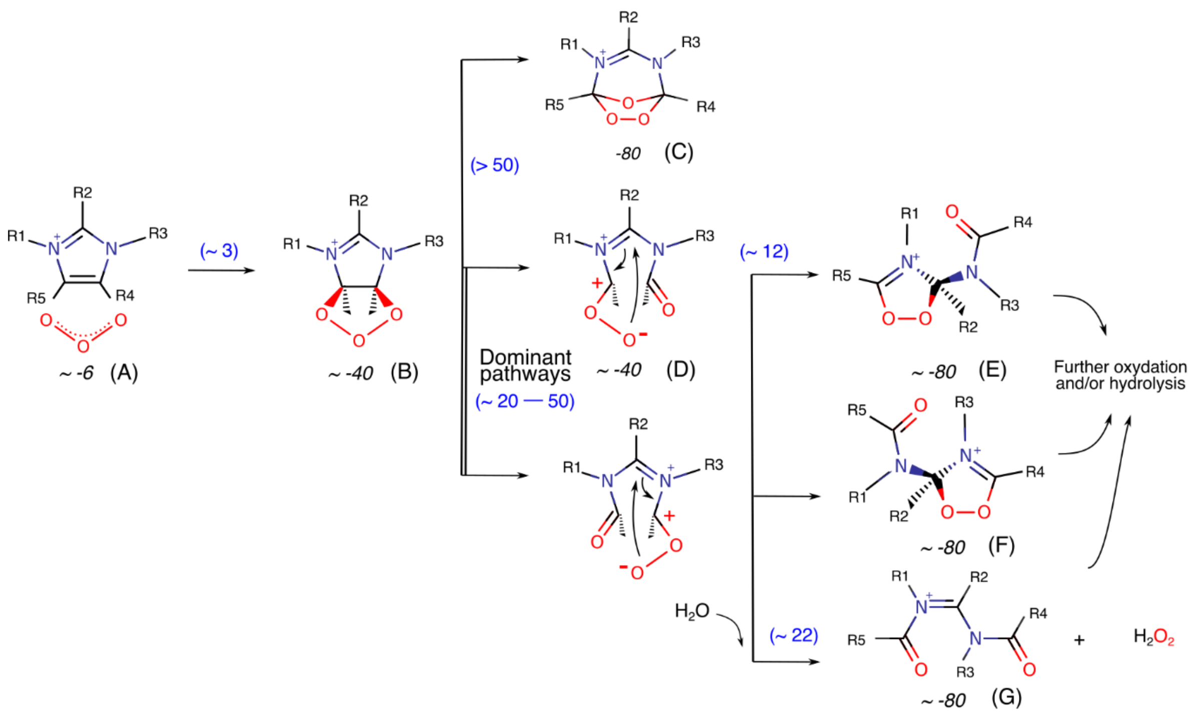

Fig. S1. Generic reaction pathway proposed for the reaction of imidazolium cations with ozone. R1 – R5 are radicals. The blue numbers are the activation energies and the italic black numbers the formation energies, both in kcal mol^{-1}.

We have also calculated the free energy barrier for the elimination of the F^- anion from the F^-@*d*4*r* (7Si,1Ge) system balanced by a H^+, to produce *d*4*r* and HF molecule. The calculation is performed using a cluster approximation for the *d*4*r*, but the coordinates of terminal hydrogen atoms are fixed to mimic the constrains of the crystal structure (from HPM-16 structure). The fluoride removal from the *d*4*r* at relatively low temperatures is very unlikely (~ 60 kcal mol^{-1}) but it could be induced by acid attack. Depending on the R1-R5 branches, some of the intermediate products could have acid hydrogens (*e.g.,* R1 = H or R3 = H in E and F). For these cases, the activation energy to extract the F from the F@D4R is reduced (~30 kcal mol^{-1}) forming HF. In addition, the H-F^-@*d*4*r* can catalyze the hydrolysis of formamide or methyl-formamide to generate NH_3, formic acid, or acetic acid. Depending on the activation energy, NH_4^+ could be stabilized by the F^-@*d*4*r* preventing the formation of H-F^-@*d*4*r* complexes, even in strong oxidation conditions. We showed this in Fig. S2-top and -middle, for the imidazolium cation (a *toy* example which we used to rationalize the degradation of the Im ring) and for 123TMI, respectively.

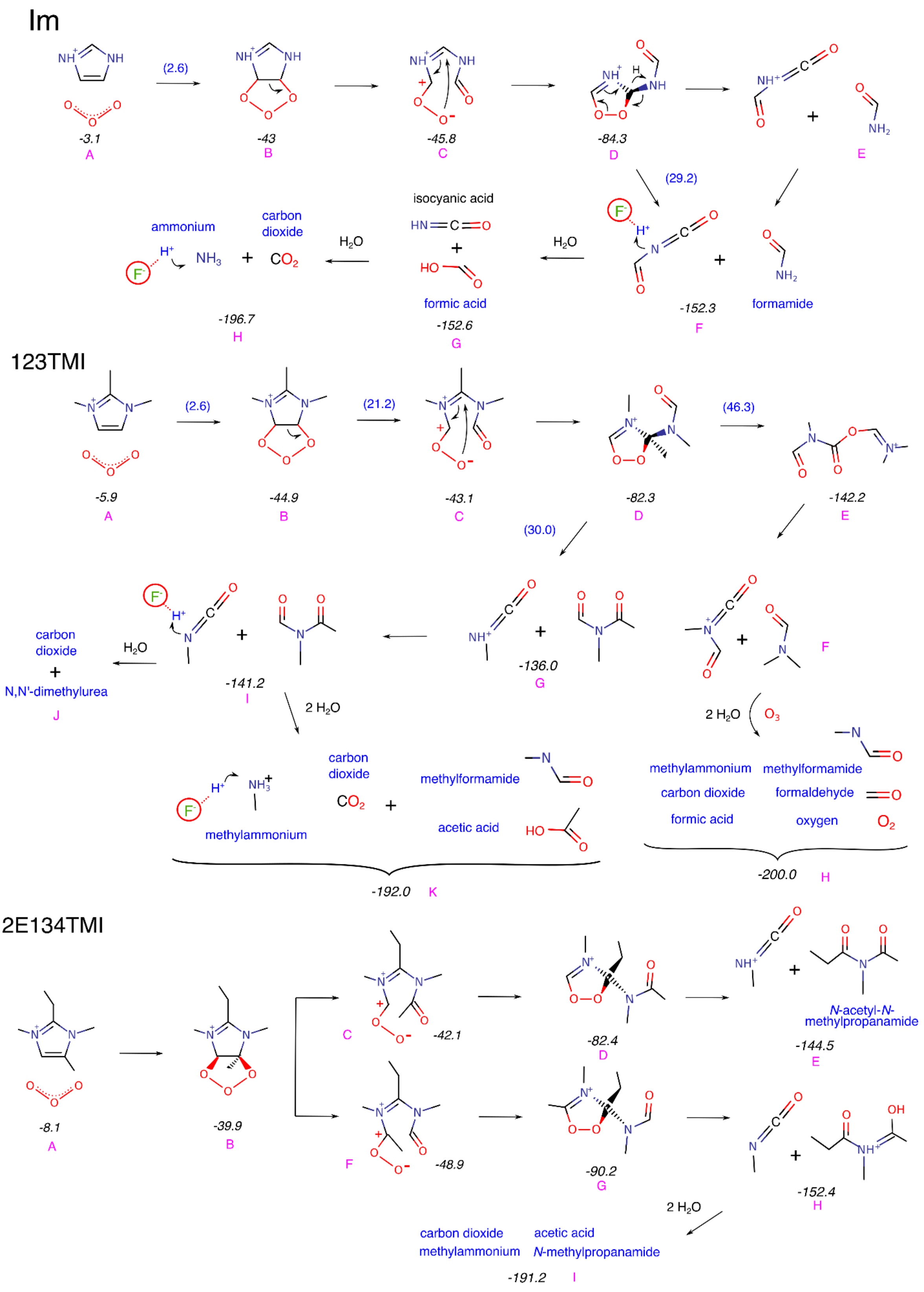

Fig. S2. Reaction pathways proposed for the reaction of Im (top), 2E134TMI (middle), and 123TMI (bottom) with O_3. The blue numbers are the activation energies and the italic black numbers the

formation energies, both in kcal mol^{-1}. The F^{-}@*d*4*r* units are symbolized by a green F inside a red circle.

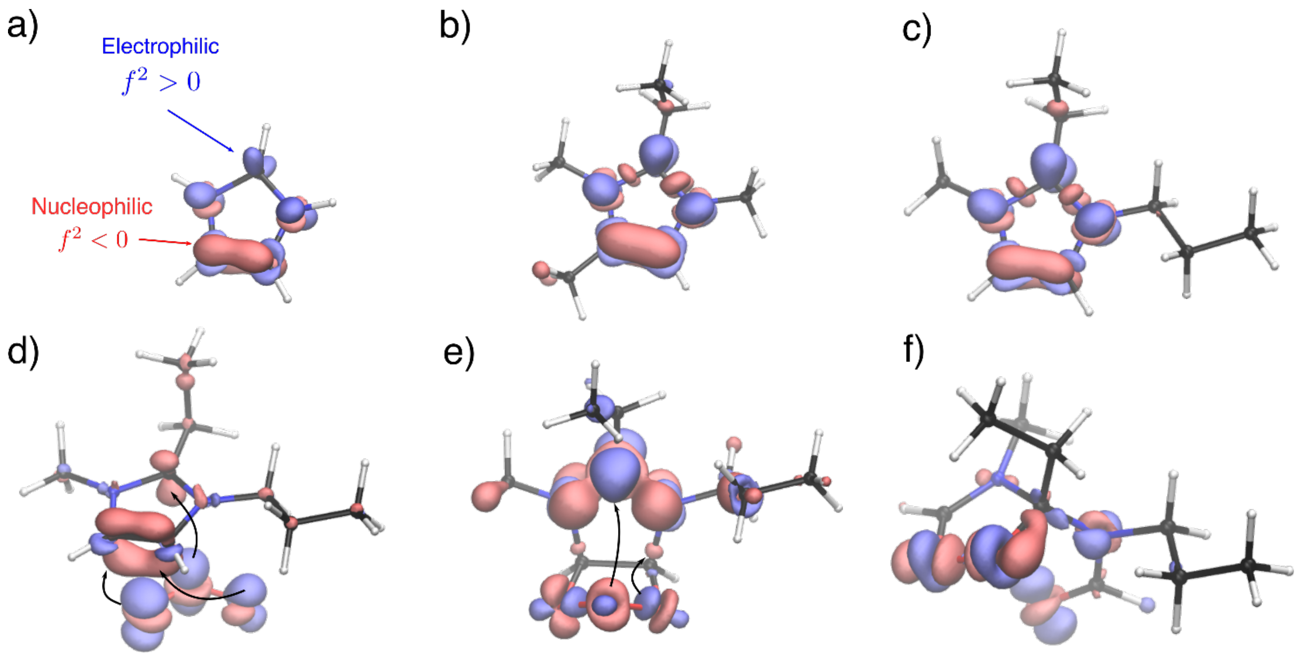

Fig. S3. Plots of the dual descriptor functions, Δ*f(r)*, as indicative of local reactivities of atoms for Δ*f(r)* < -0.05 Ha (in red color), and Δ*f(r)* > +0.05 Ha (in blue color). a) Im cation, b) 2E134TMI cation, c) 1M2E3nPrIM cation, d) the stabilizing and destabilizing interactions (black arrows) between the 1M2E3nPrIM cation and the O_3 molecule (**Fig.7-A**), e) adduct formation and stabilizing interactions (this species is very reactive towards N-C bonds and O-O bonds), f) the ring is finally broken, and the formed species is highly reactive towards the O=, and N atoms, allowing for further degradation. Some of the intermediate species (see for example, subfigure e) show highly reactive H in some of the radicals. In this way the cation can yield its charge +1 to the medium, causing more degradation products.

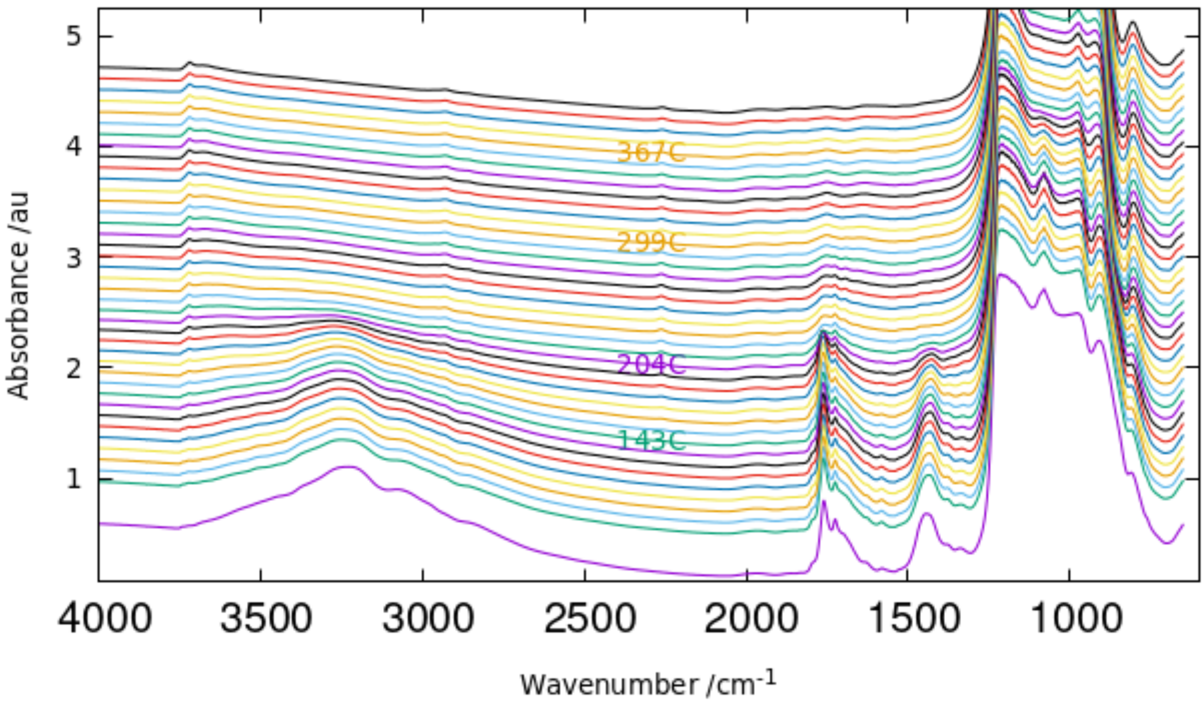

Fig. S4. FTIR study in transmission mode of the temperature programmed desorption under vacuum of a self-supporting pellet of HPM-16 after O_3 treatment at 100 ºC for 20 h. From bottom: RT, 80 ºC, and the spectra taken every 5 min during the thermal heating from 80 ºC to 400 ºC at 2 ºC/min. Some spectra are labeled with the approximate temperature.

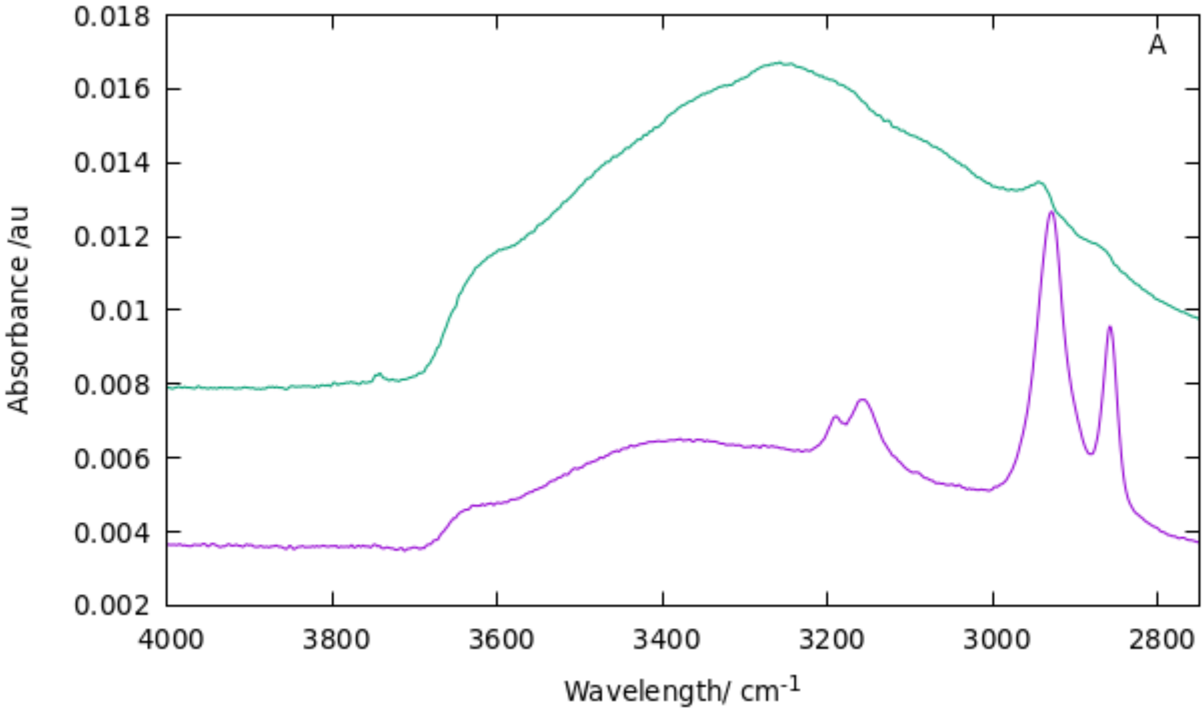

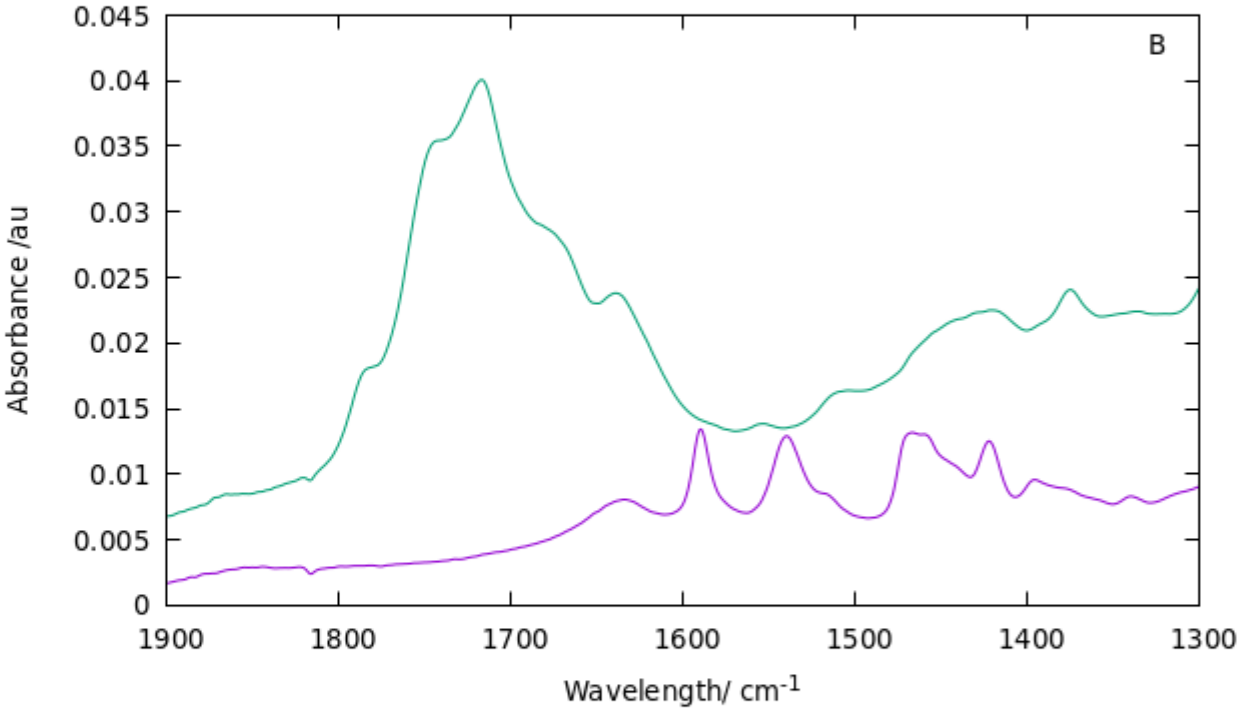

Fig. S5. ATR-FTIR spectra of HPM-7 in the OH and CH stretching (A) and imidazole ring (B) stretching regions. From bottom to top: as-made and after O_3 treatment at 100 °C for 20 h.

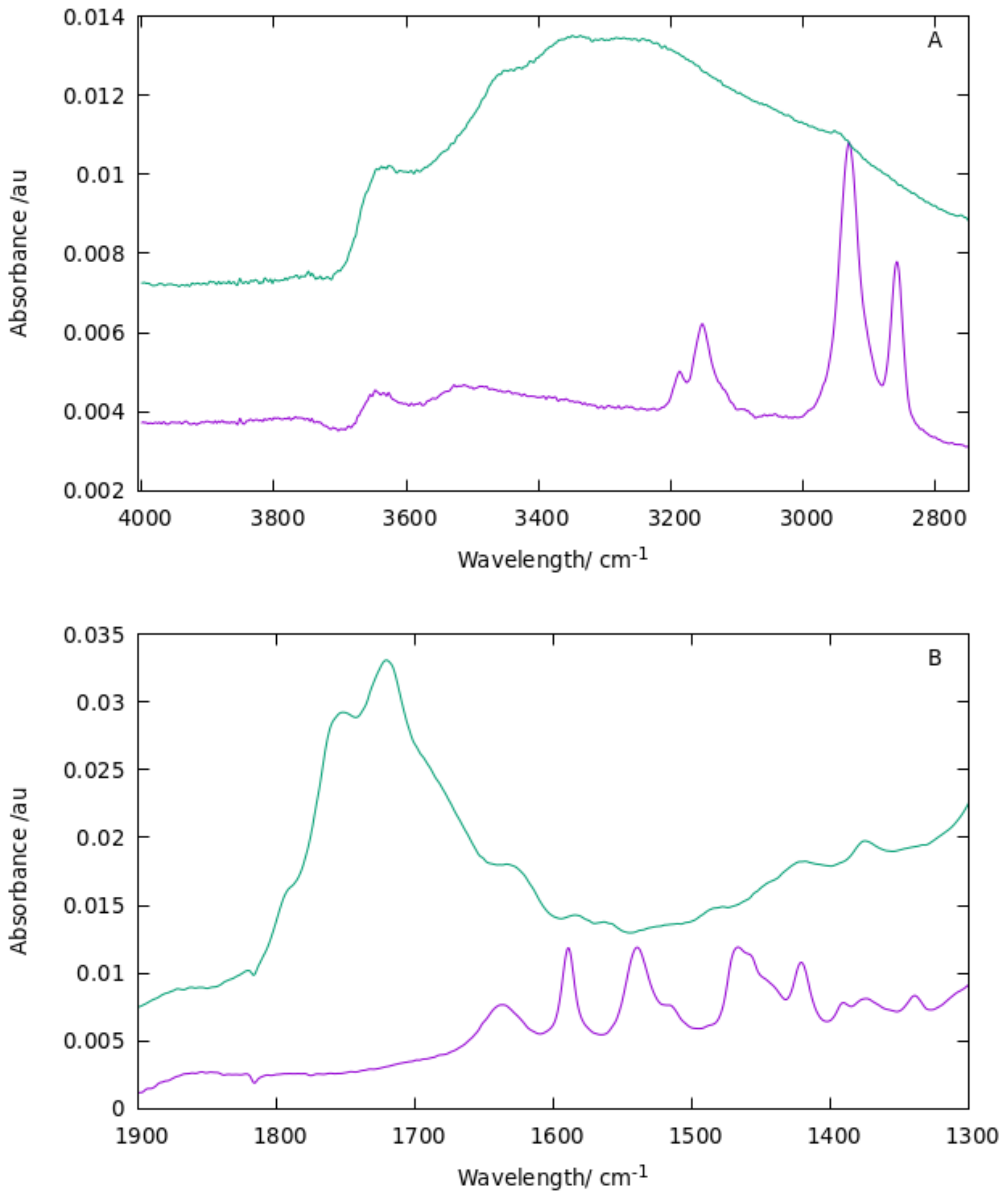

Fig. S6. ATR-FTIR spectra of HPM-8 in the OH and CH stretching (A) and imidazole ring (B) stretching regions. From bottom to top: as-made and after O_3 treatment at 100 °C for 20 h.

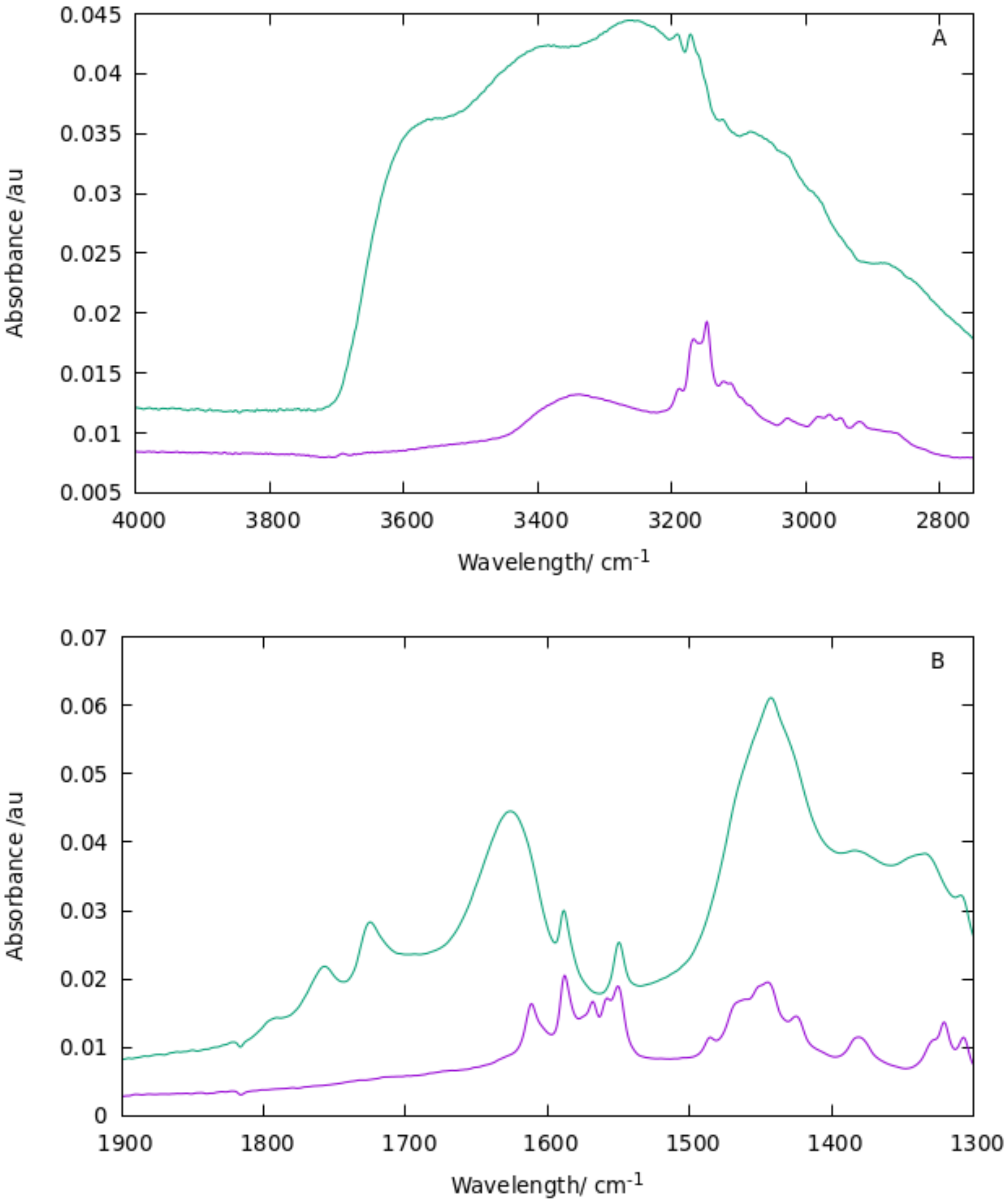

Fig. S7. ATR-FTIR spectra of HPM-14 in the OH and CH stretching (A) and imidazole ring (B) stretching regions. From bottom to top: as-made and after O_3 treatment at 100 °C for 20 h.

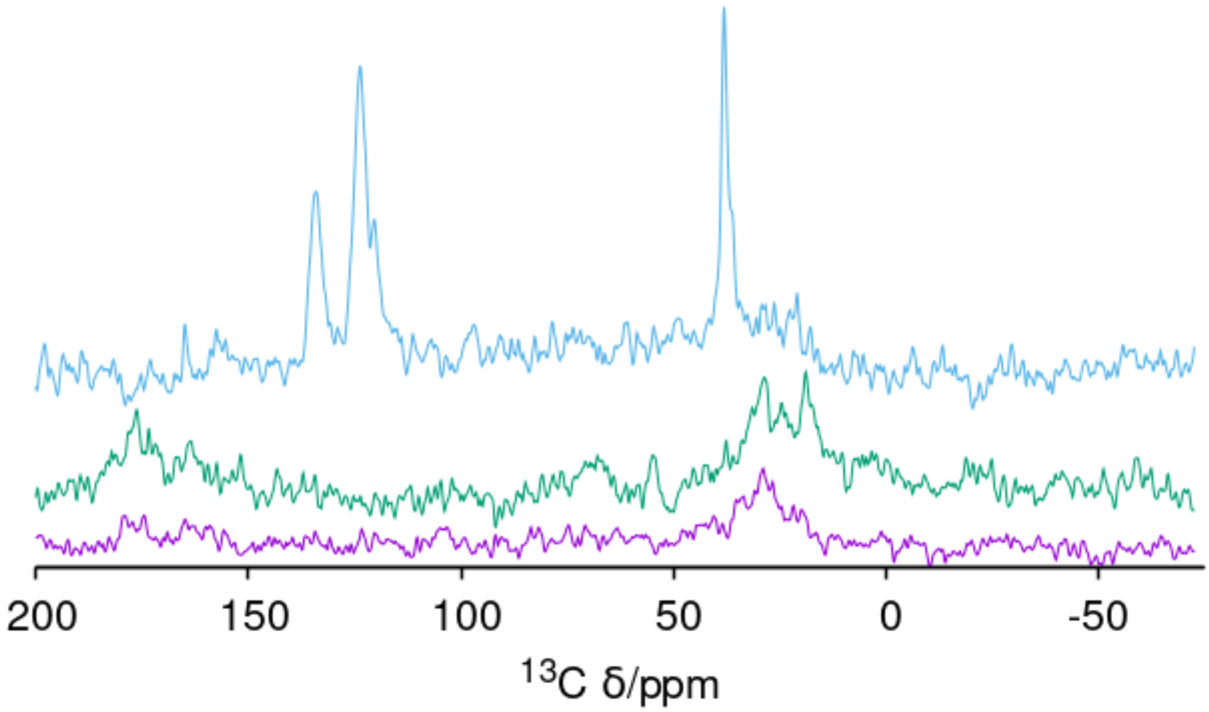

Fig. S8. ^{13}C MAS NMR spectra of (from bottom) HPM-7, HPM-8 and HPM-14 after O_3 treatment at 100 °C for 20 h.

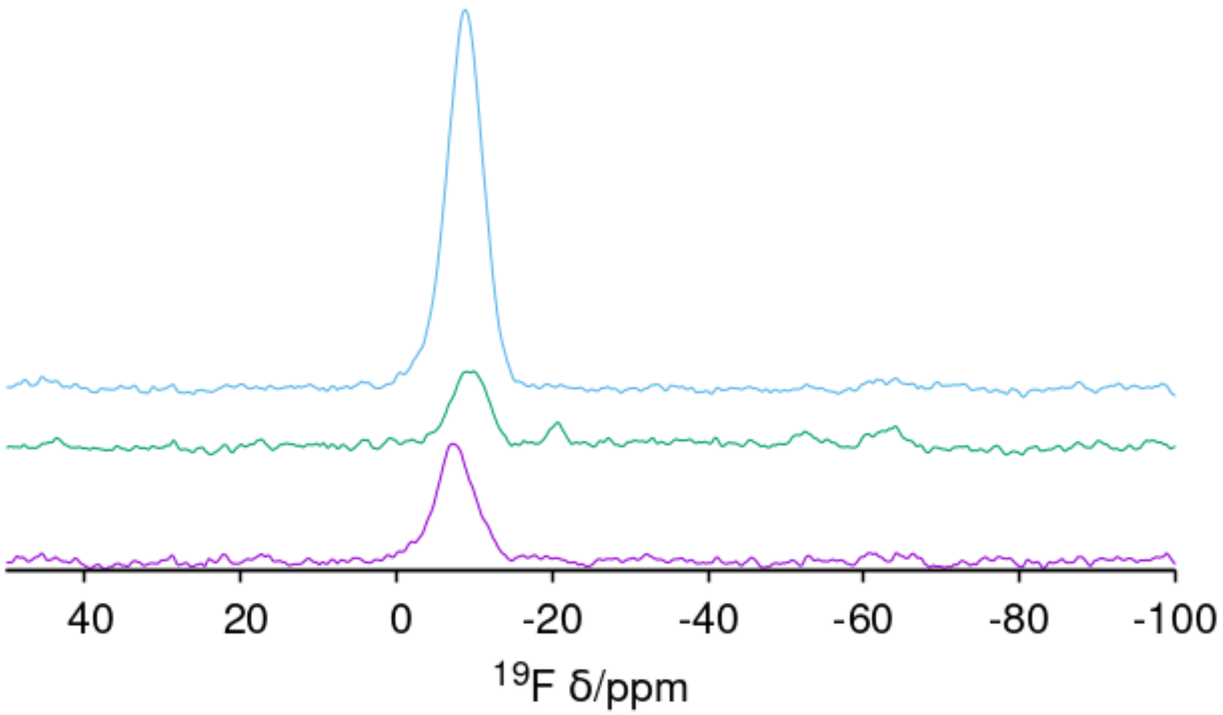

Fig. S9. ^{19}F MAS NMR spectra of (from bottom) HPM-7, HPM-8 and HPM-14 after 20 h O_3 treatment at 100 ºC, demonstrating fluoride retention within *d*4*r* units during the treatment.

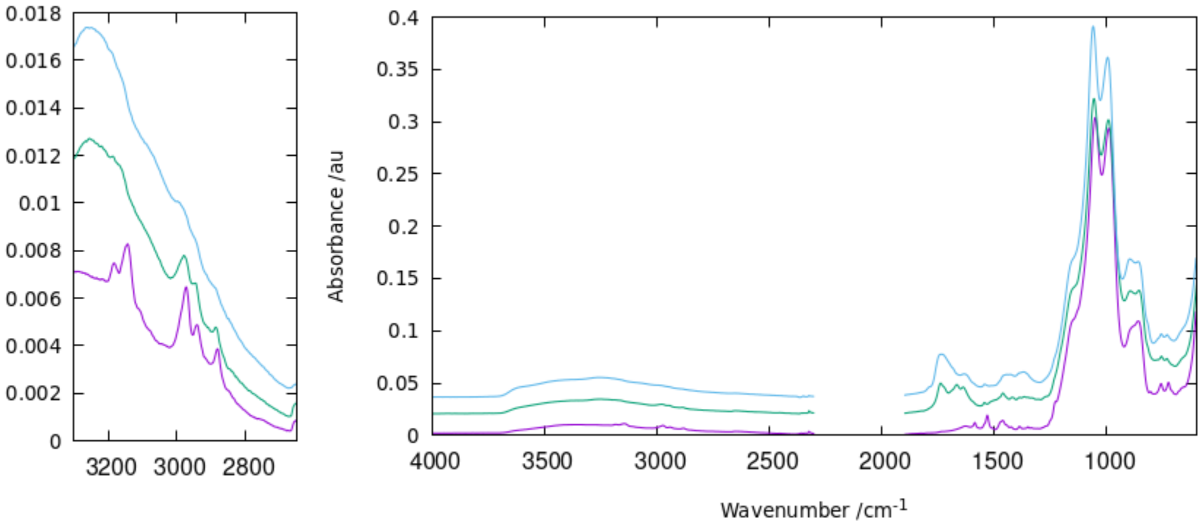

Fig. S10. ATR-FTIR spectra of **-SYT** samples (from bottom): as-made and after O_3 treatment at 100 ^{o}C for 6 and 20 h. The spectra at the left is an enlargement of the C-H stretching region.

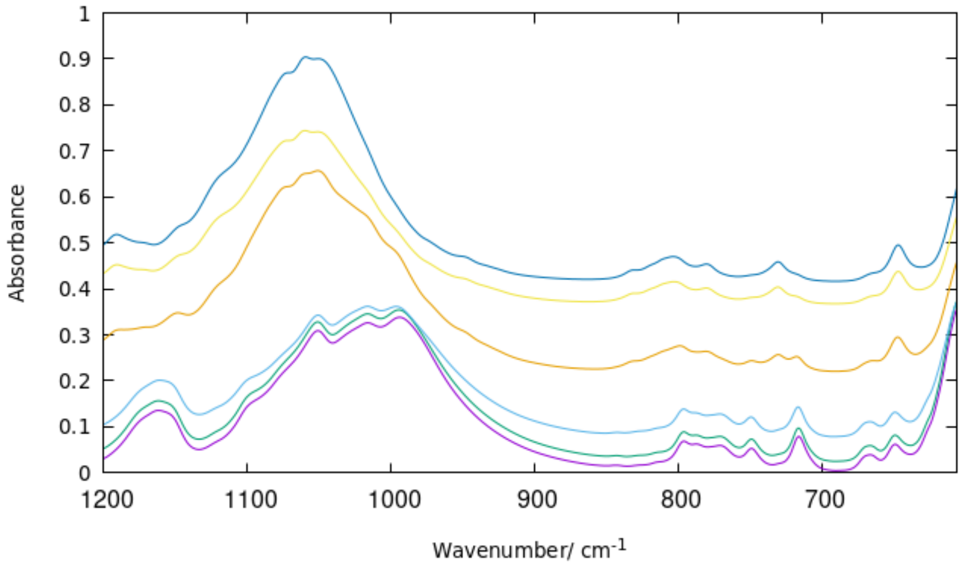

Fig. S11. Spectra in the region of framework vibrations of pure silica **STW** zeolite synthesized using 2E134TMI (from bottom): as-made and after treatments in O_3 at 100 ^oC for 20 h, 150 ^oC for 20 h, and 200 ^oC for 24 h, 48 h and 72 h.

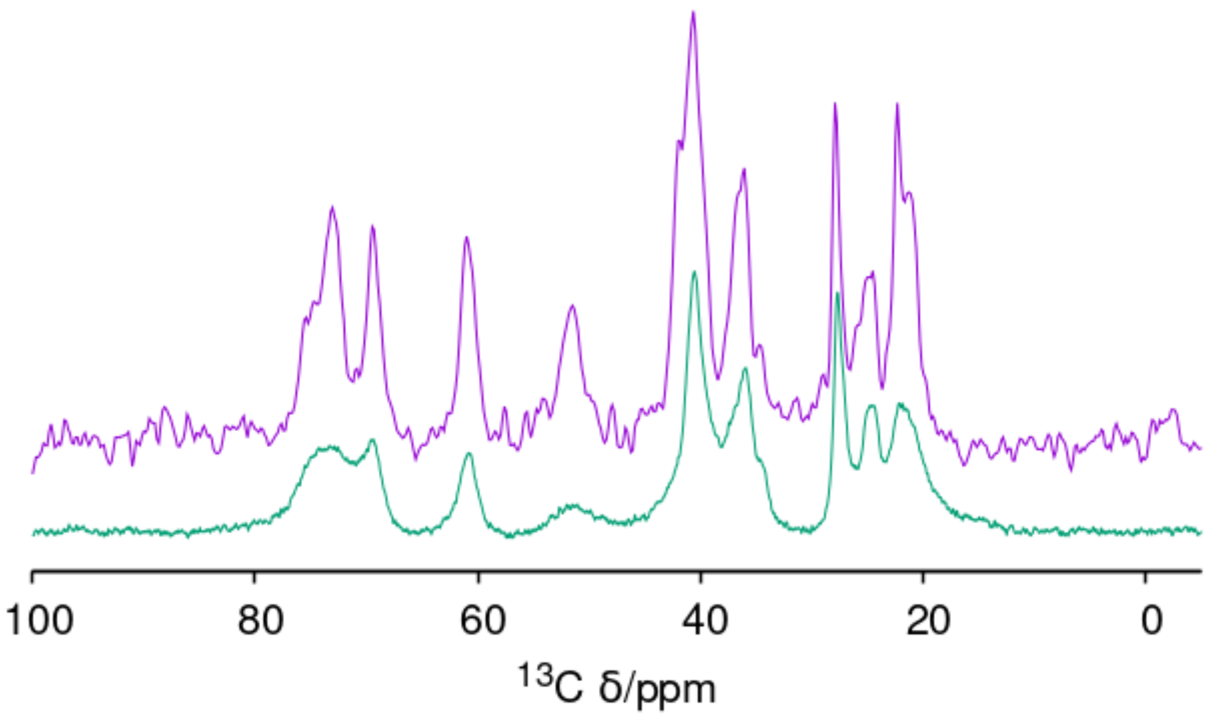

Fig. S12. ^{13}C MAS NMR spectra of pure silica **ISV**: as-made (bottom) and after O_3 treatment at 180 °C for 84 h. The difference in resolution corresponds to the different spinning speed (5 kHz and 10 kHz for bottom and top, respectively).

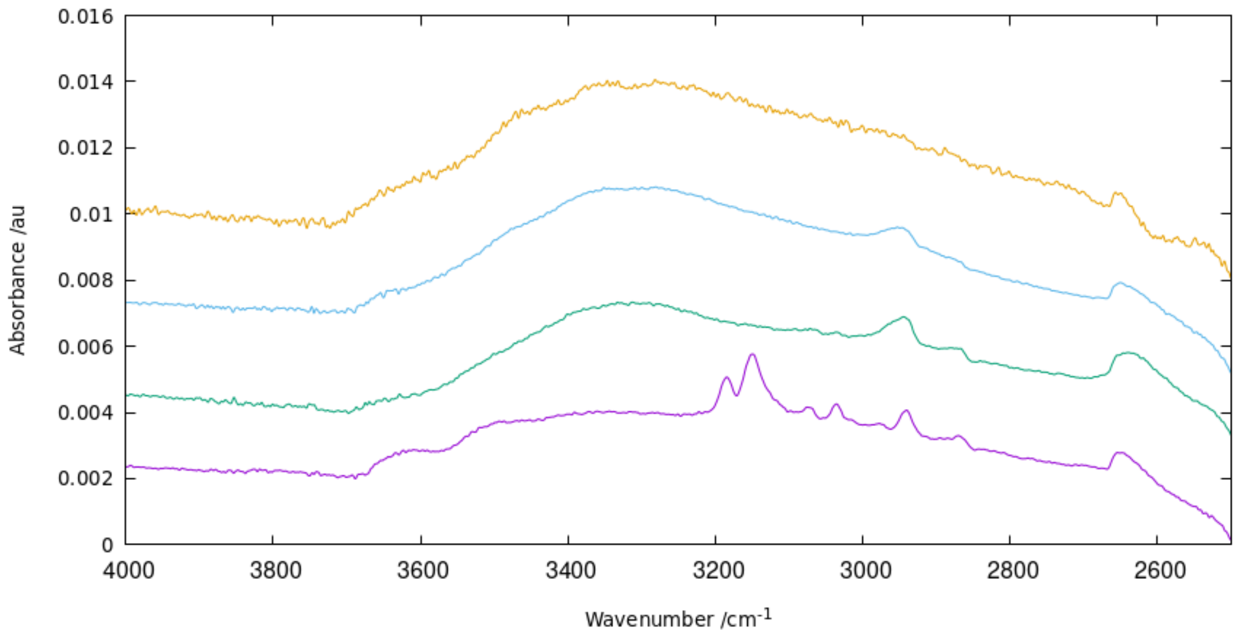

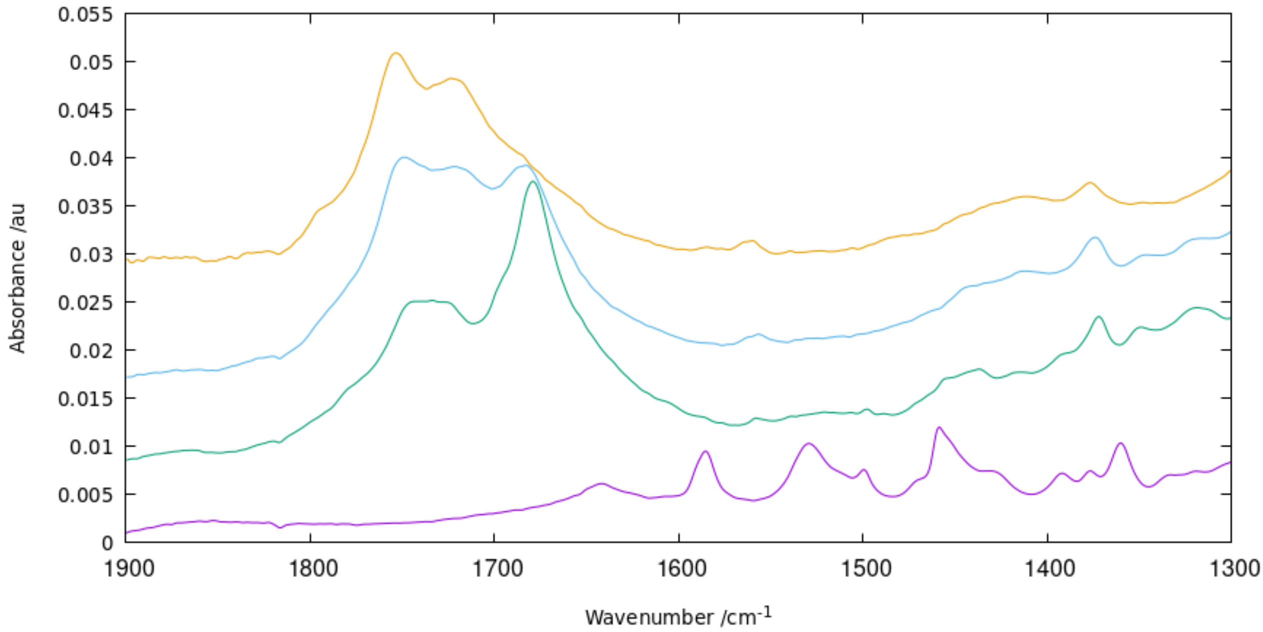

Fig. S13. ATR-FTIR spectra of silica Beta synthesized with 4bBnMI. From bottom: as-made and after O_3 treatment at 100 °C for 5 min, 2 h and 20 h.

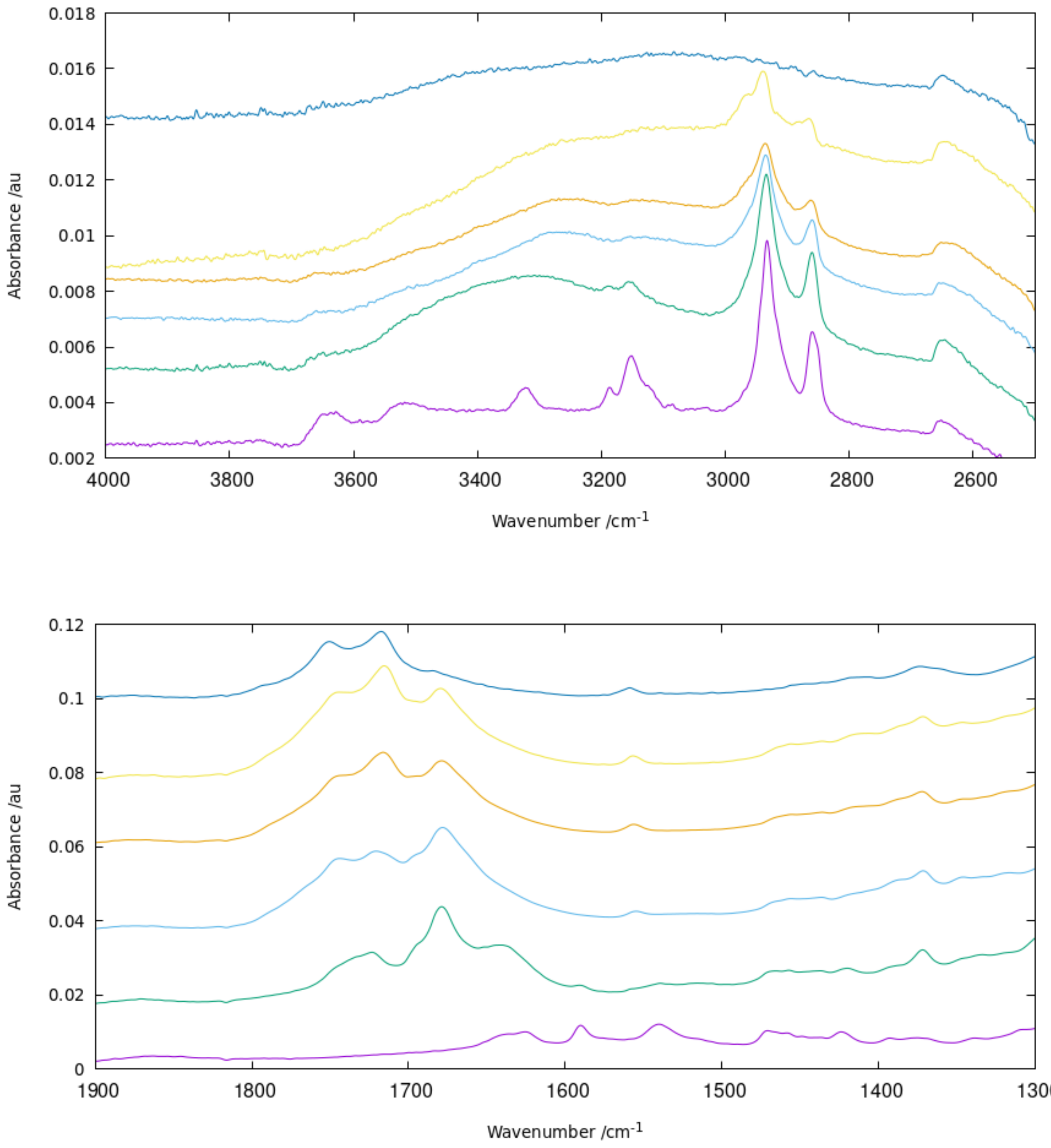

Fig. S14. ATR-FTIR spectra of silica Beta synthesized with 8bDMI. From bottom: as-made and after O_3 treatment at 100 °C for 5 min, 2 h, 20 h and 40 h and after treatment at 100 °C for 43 h followed by 150 °C for 6 h.

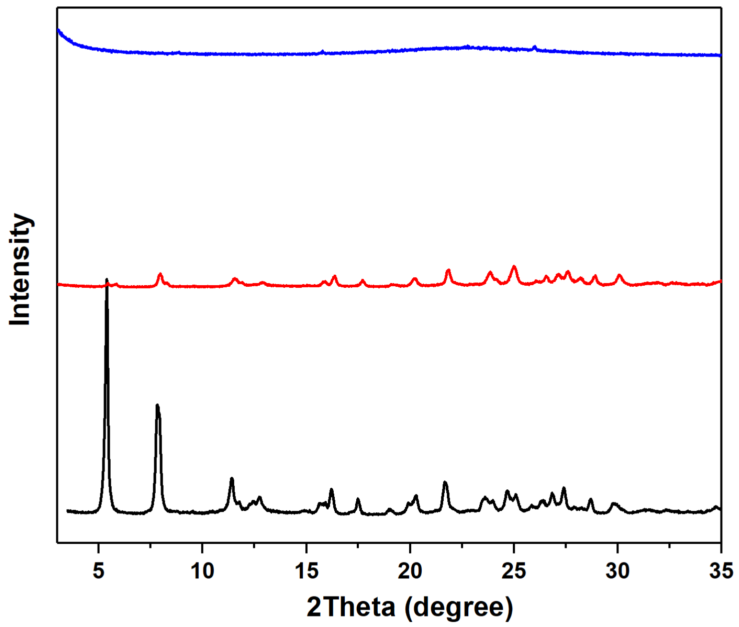

Fig. S15. PXRD patterns of HPM-14: (from bottom to top) as-synthesized, after ozone-treatment, and after degermanation.

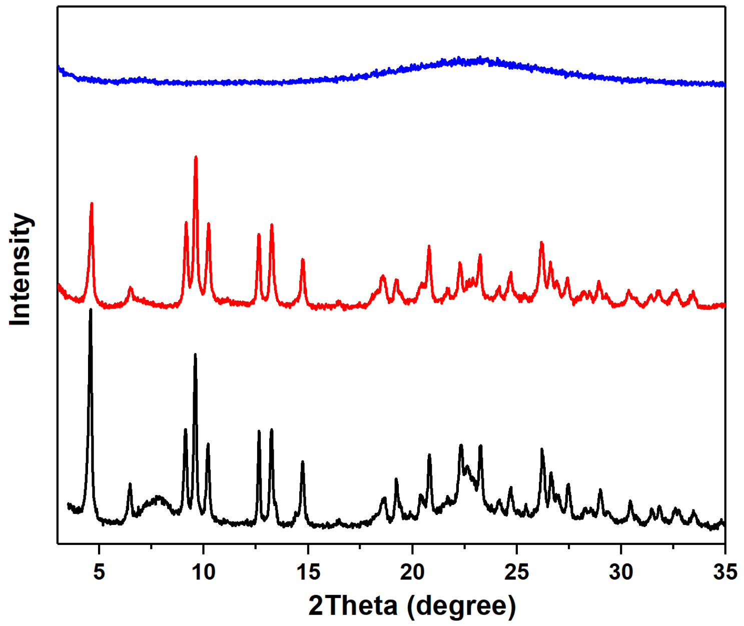

Fig. S16. PXRD patterns of SYSU-3: (from bottom to up) as-synthesized, after ozone-treatment, and after degermanation.

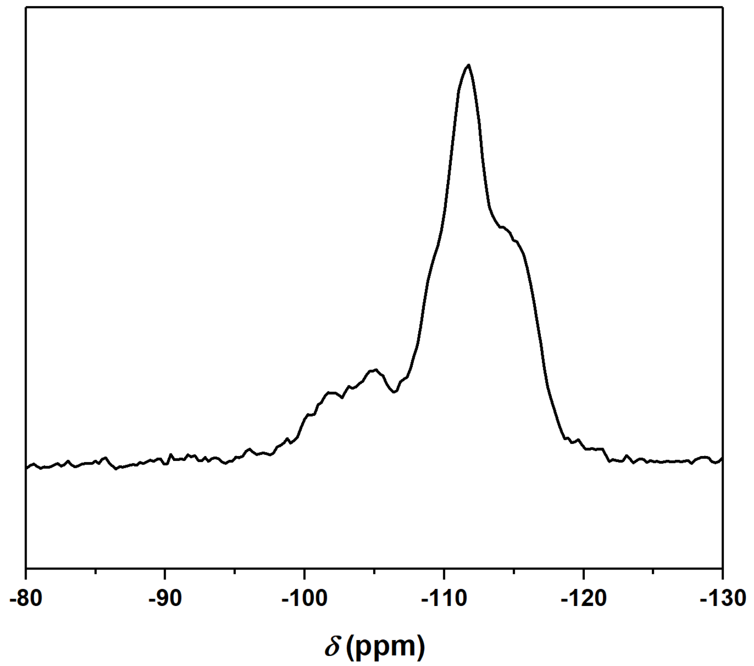

Fig. S17. ^{29}Si MAS NMR spectrum of the quasi pure-silica zeolite HPM-8 after final run of degermanation.

REFERENCES

(1)Parr, R. G.; Yang, W. Density functional approach to the frontier-electron theory of chemical reactivity. *J. Amer. Chem. Soc.* **1984**, 106, 4049–4050.

(2)Morell, C.; Grand, A.; Toro-Labbé, A. New Dual Descriptor for Chemical Reactivity. *J. Phys. Chem. A* **2004**, 109, 205–212.

(3)Morell, C.; Grand, A.; Toro-Labbé, A. Theoretical support for using the Δf(r) descriptor. *Chem. Phys. Lett.* **2006**, 425, 342–346.

(4)Martínez-Araya, J. I. Why is the dual descriptor a more accurate local reactivity descriptor than Fukui functions? *J. Math. Chem.* **2014**, 53, 451–465.
